# Supplementary material for: Catalyzing rapid discovery of gold-precipitating bacterial lineages with university students
Source: PeerJ. 2020 Apr 14;8:e8925. doi: 10.7717/peerj.8925 (PMC7164421; doi:10.7717/peerj.8925)
Supplement: Supplemental Information 2 [file peerj-08-8925-s002.docx]

**Supplemental Data S1:** Primers and probes used for real-time detection of *Delftia* and Sanger DNA sequencing.

| **Supplemental Data S1: Primers and Probes for *Delftia*** | | |
| --- | --- | --- |
| **Description** | **Sequences** | **GenBank** |
| *Delftia* gold biomineralization gene qPCR | **Seq1**, forward:  5’ ATGGCATGGGTCTATCTGTTG 3’ | CP000884.1 |
|  | **Seq2**, reverse:  5’ TCATGGTGACGATGGTGATG 3’ |  |
|  | **Seq3**, double-quenched probe:  5’ /56-FAM/ TCTGGGCGT / ZEN / TCTCGATGAAGCAAT /3IABkFQ/ 3’ |  |
| *Delftia* unique toxin-antitoxin sequence qPCR | **Seq4**, forward: 5’ GCTGCTCGAAGTGGTCTG 3’ | CP000884.1 |
|  | **Seq5**, reverse: 5’ ATGGTGATGGCGCTTGG 3’ |  |
|  | **Seq6**, double-quenched probe:  5’ /56-FAM/ CGATGAAGC / ZEN / AATCGCACGGCTT  /3IABkFQ/ 3’ |  |
| *Delftia* gold gene Sanger sequencing | **Seq7**, forward: 5’ AGATGTCCTGGATGTGGCT 3’ | CP000884.1 |
|  | **Seq8**, reverse: 5’ CTCATCAACATGTACGGCA 3’ |  |
