# Supplementary material for: Catalyzing rapid discovery of gold-precipitating bacterial lineages with university students
Source: PeerJ. 2020 Apr 14;8:e8925. doi: 10.7717/peerj.8925 (PMC7164421; doi:10.7717/peerj.8925)
Supplement: Supplemental Information 3 [file peerj-08-8925-s003.docx]

**Supplemental Data S2:** qPCR conditions

A 20 µl qPCR reaction for identifying *Delftia* within metagenomic samples was performed using 0.8 µl of 10 µM primers **Seq1** and **Seq2** and 0.4 µl of 10 µM double-quenched probe **Seq3**, 10 µl of 2X Luna Universal Probe qPCR Master Mix, 2 µl metagenomic DNA, and 6 µl deionized water for each 20 µl reaction targeting the gold gene. Additionally, primers **Seq4** and **Seq5** with double-quenched probe **Seq6** were used in separate 20 µl reactions for confirmation with the CP sequence, using identical volumes and concentrations. New England BioLabs Luna Universal Probe qPCR Master Mix reagents were prepared with an Eppendorf epMotion 5075 liquid handler. A Bio-Rad CFX Connect was used to amplify and detect fluorescence according to Luna Universal Probe protocol. Initial denaturation was at 95°C for 60 seconds, followed by 40 cycles of denaturation at 95°C for 15 seconds and extension at 60°C for 30 seconds. An 8-point ten-fold dilution standard curve was used starting at 40 pg/µl concentration for the gold gene standard and 30 pg/µl for the CP sequence standard.

“Gold Gene” Standard Curve Calculations Beginning with 40 pg/µl Standard

| **Dilution #** | **Size in bp** | **6.6x10^11 ng/mole*bp; per base pair** | **mass in ng/mole** | **mass added in ng** | **number moles** | **x Avogadro's = number of copies** |
| --- | --- | --- | --- | --- | --- | --- |
| 1 | 106 | 6.60E+11 | 7.00E+13 | 0.00008 | 1.14E-18 | 6.89E+05 |
| 2 | 106 | 6.60E+11 | 7.00E+13 | 0.000008 | 1.14E-19 | 6.89E+04 |
| 3 | 106 | 6.60E+11 | 7.00E+13 | 0.0000008 | 1.14E-20 | 6.89E+03 |
| 4 | 106 | 6.60E+11 | 7.00E+13 | 0.00000008 | 1.14E-21 | 6.89E+02 |
| 5 | 106 | 6.60E+11 | 7.00E+13 | 0.000000008 | 1.14E-22 | 6.89E+01 |
| 6 | 106 | 6.60E+11 | 7.00E+13 | 0.0000000008 | 1.14E-23 | 6.89E+00 |
| 7 | 106 | 6.60E+11 | 7.00E+13 | 0 | 1.14E-24 | 6.89E-01 |
| 8 | 106 | 6.60E+11 | 7.00E+13 | 0 | 1.14E-25 | 6.89E-02 |

CP Gene Standard Curve Calculations Beginning with 30 pg/µl Standard

| **Dilution #** | **Size in bp** | **6.6x10^11 ng/mole*bp; per base pair** | **mass in ng/mole** | **ng mass added** | **number moles** | **x Avogadro's = number of copies** |
| --- | --- | --- | --- | --- | --- | --- |
| 1 | 78 | 6.60E+11 | 5.15E+13 | 0.00006 | 1.17E-18 | 7.02E+05 |
| 2 | 78 | 6.60E+11 | 5.15E+13 | 0.000006 | 1.17E-19 | 7.02E+04 |
| 3 | 78 | 6.60E+11 | 5.15E+13 | 0.0000006 | 1.17E-20 | 7.02E+03 |
| 4 | 78 | 6.60E+11 | 5.15E+13 | 0.00000006 | 1.17E-21 | 7.02E+02 |
| 5 | 78 | 6.60E+11 | 5.15E+13 | 0.000000006 | 1.17E-22 | 7.02E+01 |
| 6 | 78 | 6.60E+11 | 5.15E+13 | 0.0000000006 | 1.17E-23 | 7.02E+00 |
| 7 | 78 | 6.60E+11 | 5.15E+13 | 0 | 1.17E-24 | 7.02E-01 |
| 8 | 78 | 6.60E+11 | 5.15E+13 | 0 | 1.17E-25 | 7.02E-02 |
