# Supplementary material for: Catalyzing rapid discovery of gold-precipitating bacterial lineages with university students
Source: PeerJ. 2020 Apr 14;8:e8925. doi: 10.7717/peerj.8925 (PMC7164421; doi:10.7717/peerj.8925)
Supplement: Supplemental Information 4 [file peerj-08-8925-s004.docx]

**Supplemental Data S3:** *Delftia* “gold” gene PCR amplification conditions.

A PCR reaction prior to sequencing was performed using **Seq7** and **Seq8** primers in a 50µl reaction volume consisting of 0.25µl each of 100µM forward and reverse primers, 2µl template DNA, 24µl NEB Q5 Hot Start High-Fidelity Master Mix, and 23.5µl deionized water. PCR was done with a Bio-Rad PCR machine, programmed for 30s initial denaturation at 98°C with 35 cycles of 10s denaturation at 98°C, 30s annealing at 65°, 20s extension at 72°C, and 2min final extension at 72°C. PCR products were verified by gel electrophoresis at 140V on a 2% agarose gel in 1X TBE buffer with a 1kb ladder. The gel was stained for 5 min in 1X gel red, destained for 5 min in TBE buffer, and visualized using a Bio-Rad transilluminator.
