# Supplementary material for: Catalyzing rapid discovery of gold-precipitating bacterial lineages with university students
Source: PeerJ. 2020 Apr 14;8:e8925. doi: 10.7717/peerj.8925 (PMC7164421; doi:10.7717/peerj.8925)
Supplement: Supplemental Information 5 [file peerj-08-8925-s005.docx]

**Supplemental Data S4:** Sequences and Clustal alignment of unique “gold” gene in *Delftia* species and strains.

>CP019171.1:4965063-4966107 Delftia acidovorans isolate ANG1, complete genome
AGATGTCCTGGATGTTGGCTGCGCCACCGGGCACCGCCTGCGCAATGCGAGCAATCTCCTCCTCATCCAG
CGCCACCAGGGTCAGCATGTCCGGCGTGATCGCCGTGCAGCCTTCGGGGATGCCGTTGGGCGGCACATCG
ATCTCGCCGGCCACCTGCTCCCCTTGGTCCCCCTGCTGCGCCTGCAACACTGCCTGCGCAAACTCCGCCA
GCCTCGGGTGCTGGAACAGCGTGCGCACCTGCACGCGCAGGCCCTGGGCGCGCACGCGCTCCAGCAGGCC
CAGGGCCAGCAGCGAATGCCCGCCCAGCTCGAAGAAGCCGTCCTGCCGGCCCACGCGATCCACGCCCAGC
ACCTCGGCCCAGATCGTCGCCAGCGTTTCCTCCAACTCGCCCTGCGGTGCCTCGTATTGCTGGGCGCTGA
TCATCTCCGGCTCGGGCAGCGCCTTGCGGTCCACCTTGCCGTTGGCTGTCAGCGGCAGGGCTTCGAGCAC
GACGATGGCCGAGGGCACCATGTAGTCGGGCAGCGCCTGGCCCAGGCGCTGCTTGAGCTGGCTTTCCTCC
ACCGCGTCACGCAGGGAGACATAGGCGATCAGCCTTGCACCCTCCTTGGCCAGCACCACCGCCTCACGCA
CTTCAGGCTGGGCCAGCAGCTGCGACTGCACCTCGCCCAGTTCGATGCGGAAGCCCCGGATCTTGACCTG
CTGGTCGGCACGACCCAGGTATTCGAGTTCGCCCTGTGCACTCCAGCGCACCAGATCGCCCGTGCGGTAC
AGGCGATCGCCTGCCTGTGTGAAGGGATTGGCGATAAAGCGCTCGGCGCTCAGGCCAGCGCGATTCAGGT
AGCCGCGTGCCAGTCCTTCGCCCGCCACGTACAGCTCCCCGGCCACGCCCTGCGGCAGCAGGTTCAGGCT
GCCATCGAGCACGTACAGGCCCAGGTCCGGAATAGCCACGCCAACGGGGCTGCGGCCACCATCCAAATCC
GCCTTGGTGATCTGACGGTACGTCACATGCACCGTGGTCTCGGTGATGCCGTACATGTTGATGAG

>CP000884.1:5233319-5234363 Delftia acidovorans SPH-1, complete genome
AGATGTCCTGGATGTTGGCTGCGCCACCGGGCACCGCCTGCGCAATGCGAGCAATCTCCTCCTCATCCAG
CGCCACCAGGGTCAGCATGTCCGGCGTGATCGCCGTGCAGCCTTCGGGGATGCCGTTGGGCGGCACATCG
ATCTCGCCGGCCACCTGCTCCCCTTGGTCCCCCTGCTGCGCCTGCAACACTGCCTGCGCAAACTCCGCCA
GCCTCGGGTGCTGGAACAGCGTGCGCACCTGCACGCGCAGGCCCTGGGCGCGCACGCGCTCCAGCAGGCC
CAGGGCCAGCAGCGAATGCCCGCCCAGCTCGAAGAAGCCGTCCTGCCGGCCCACGCGATCCACGCCCAGC
ACCTCGGCCCAGATCGTCGCCAGCGTTTCCTCCAACTCGCCCTGCGGTGCCTCGTATTGCTGGGCGCTGA
TCATCTCCGGCTCGGGCAGCGCCTTGCGGTCCACCTTGCCGTTGGCTGTCAGCGGCAGGGCTTCGAGCAC
GACGATGGCCGAGGGCACCATGTAGTCGGGCAGCGCCTGGCCCAGGCGCTGCTTGAGCTGGCTTTCCTCC
ACCGCGTCACGCAGGGAGACATAGGCGATCAGCCTTGCACCCTCCTTGGCCAGCACCACCGCCTCACGCA
CTTCAGGCTGGGCCAGCAGCTGCGACTGCACCTCGCCCAGTTCGATGCGGAAGCCCCGGATCTTGACCTG
CTGGTCGGCACGACCCAGGTATTCGAGTTCGCCCTGTGCACTCCAGCGCACCAGATCGCCCGTGCGGTAC
AGGCGATCGCCTGCCTGTGTGAAGGGATTGGCGATAAAGCGCTCGGCGCTCAGGCCAGCGCGATTCAGGT
AGCCGCGTGCCAGTCCTTCGCCCGCCACGTACAGCTCCCCGGCCACGCCCTGCGGCAGCAGGTTCAGGCT
GCCATCGAGCACGTACAGGCCCAGGTCCGGAATAGCCACGCCAACGGGGCTGCGGCCACCATCCAAATCC
GCCTTGGTGATCTGACGGTACGTCACATGCACCGTGGTCTCGGTGATGCCGTACATGTTGATGAG

>CP022656.1:223076-224129 Delftia acidovorans strain RAY209 genome
AGATGTCCTGGATGTTGGCTGCGCCACCGGGCACCGCCTGCGCAATGCGTGCAATCTCATCCTCATCCAG
CGCCACCAGGGTCAGCATGTCCGGCGTGATCTCCGTGCAGCCTTCGGGGATGCCGTTGGGCGGCACATCG
ATCTCGCCGGCCACCTGCGCCCCTGGCTCACCCTGCTGATCCTGCAGCACCGCCTGCGCAAACTCCGCCA
ACCTCGGGTGCTGGAACAGCGTGCGCACCTGCACGCGCAGGCCCTGGGCGCGCACGCGCTCCAGCAGGCC
CAGGGCGAGCAGAGAATGCCCGCCCAGCTCGAAGAAGCCGTCCTGCCGTCCCACGCGCTCCACGCCCAGC
ACCTCGGCCCAGATCTTCGCCAGCGTTTCTTCGAGTTCACCTTGCGGTGCCTCGTATTCCTGCGCACTCG
CCATCTCCGGCTCGGGCAGCGCCTTGCGGTCCACCTTGCCGTTGGCAGTCAGCGGCAGGGCGTCAAGCGC
GACGATGGCCGAGGGCACCATGTAGTCGGGCAGCGCCTGGCCCAGCCGGTCCTTGAGCCAACCGTCTTCG
ACCGGCGAATTCAGCGAGACATAGGCCACCAATCGCGCCCCGCCTGTGCTTTCCTTGGCCAGCACCACGG
CCTCGCGCACCTCGGGCTGGGCCAGCAGCTGCGACTGCACCTCGCCCAGCTCGATGCGGAAGCCCCGGAT
CTTGACCTGCTGGTCGGCACGGCCCAGGTATTCGAGTTCGCCCTGAGCGTTCCAGCGCACCAGATCGCCC
GTGCGGTACAGGCGATCGCCTGCCTGTGTAAAGGGATTGGCGATGAAGCGCTCGGCGCTCAGACCGGCGC
GGTTCAGGTAACCACGTGCCAGGCCCTCACCCGCCACATACAACTCTCCCGCCACGCCCTGCGGCAGCAG
GTTCAGGCTGCCGTCGAGCACGTACAGGCCCAGGTCCGGAATCGCCACGCCCACGGGGCTGCGTCCGCCG
TCCAGATCCTTCCGGGTGATCTCCCGGTACGTCACATGCACCGTGGTCTCGGTGATGCCGTACATGTTGA
TGAG

>CP018101.1:4891000-4892038 Delftia sp. HK171, complete genome
AGATGTCCTGGATGTTGGCTGCACCACCGGGCACGGCTGCGGCGATCCGTGCAATCTCCTGGGCGTCCAG
CGCCACCAGGGTCAGCATGGCCGGGGTGATGGCCGTGCAGTCTTCGGGAATGCCGTTGGGCGGCACCTGC
ACCTCGGGTCGAGCATCGGCCTGTTCGCCACCAAGGGCCTGCGCAAAGTCGGCCAGCCTGGGCTGAAGGA
ACAGGCTGCGCACCTGGGCCGCCAGCCCCCTGGCGCGCATGCGTTCGAGCAGCTTCAGGGCCAGCAACGA
ATGACCACCCAGCTCGAAGAAGCTGTCATGGCGTCCCACGCGCTCCACGCCCAGCACCTCGGCCCAGATG
CGCGCCAGCGCCTCTTCCAGTTCGCCCTGCGGCGCCTCGTATTGCTGGGTACTTGCCATTTCCGGTTCAG
GCAAGGCCTTGCGGTCCACCTTGCCATTGGCCGTCAACGGCAGGGCATCGAGCACCACGATGGCCGAAGG
CACCATGTAGTCAGGCAGCCCCTGACCCAGCCGCTCCTTGAGCAGCCCATCTTCGACTTCCTCGTTCAGC
GAGACATAGGCCACCAGGCGCGCCCCGCCCGGCCCCTGGCTGGCCAGCACGACGGCCTCACGCACCTCCG
GCTGCGCCAGCAGCTGGGCCTGCACTTCGCCCAGCTCGATGCGGAAGCCCCGGATCTTGACCTGCTGATC
GACCCGGCCCAGGTATTCGAGCTCGCCCTGCGCGTTCCAGCGCACCAGGTCGCCTGTGCGGTACAGCCTG
CTGCCATCGTCCGTGAAGGGGTTGGCGATGAAGCGCTCCGCGCTCAGGCCTTGCCGATTCAGGTAGCCAC
GCGCCAGGCCCGCGCCCGCAACGAACAGCTCGCCCGCCACGCCTTGCGGCAACAGGTTCAGACTGCCGTC
CAGCACGTACAGGCCCAGGTCCGGAATGGCAGTTCCCACGGGACTGCGGCCACCGTCCAAATCTGTCTTG
GTGATCTCCCGGTACGTCACATGCACCGTGGTCTCGGTGATGCCGTACATGTTGATGAG

>CP017420.1:7071774-7072827 Delftia tsuruhatensis strain CM13, complete genome
CTCATCAACATGTACGGCATCACCGAGACCACGGTGCATGTGACGTACCGGGAGATCATCCAGAAGGATC
TGGACGGCGGACGCAGCCCCGTGGGCGTGGCGATTCCGGACCTGGGCCTGTACGTGCTCGACGGCAGCCT
GAACCTGCTGCCGCAGGGTGTGGCGGGAGAGTTGTATGTGGCGGGCGAGGGCTTGGCACGCGGCTATCTC
AACAGGCAAGGACTGACGGCAGAGCGCTTCATTGCCAACCCGTTCAGCGAGACGGGCGAGCGCCTGTACC
GCACGGGCGACCTGGTGCGCTGGAGTGCACAGGGCGAACTCGAATACCTGGGTCGTGCCGACCAGCAGGT
CAAGATCCGGGGCTTCCGCATCGAGCTGGGCGAGGTGCAGTCGCAACTGCTGGCCCAGCCCGAGGTGCGC
GAGGCCGTGGTTTTGGCCAAGGAGGGTGCAAGGCTGATCGCCTATGTCTCCCTGCGTGACGCGGTGGAGG
AAAGCCAGCTCAAGCAGCGCCTGGGCCAGGCGCTGCCCGACTACATGGTGCCCTCGGCCATCGTCGTGCT
CGATGCCCTGCCTTTGACGGCCAACGGCAAGGTGGACCGCAAGGCGCTGCCCGAGCCGGAGATGGCGAGT
GCGCAGGAATACGAGGCACCGCAAGGTGAACTCGAAGAAACGCTGGCGCAGATCTGGGCCGAGGTGCTGG
GCGTGGAGCGCGTGGGACGGCAGGACGGCTTCTTCGAGCTGGGCGGGCATTCGCTGCTGGCCCTGGGCCT
GCTGGAGCGCGTGCGCGCCCGGGGCCTGCGCGTGCAGGTGCGCACGCTGTTCCAGCACCCGAGGCTGGCG
GAGTTTGCGCAGGCGGTGCGGCAGGAGCAGCAGGGTGAGCAGGGTGAGCAAGGTGCGCAGGTGGCCGGCG
AGATCGACGTGCCGCCCAATGGCATCCCCGAAGGCTGCACGGCGATCACGCCGGACATGCTGACCCTGGT
GGCGCTGGATGAAGATGAGATTGCACGCATTGCCGCTGCGGTGCCCGGTGGCGCAGCCAACATCCAGGAC
ATCT

(Reverse Complement):
AGATGTCCTGGATGTTGGCTGCGCCACCGGGCACCGCAGCGGCAATGCGTGCAATCTCATCTTCATCCAGCGCCACCAGGGTCAGCATGTCCGGCGTGATCGCCGTGCAGCCTTCGGGGATGCCATTGGGCGGCACGTCGATCTCGCCGGCCACCTGCGCACCTTGCTCACCCTGCTCACCCTGCTGCTCCTGCCGCACCGCCTGCGCAAACTCCGCCAGCCTCGGGTGCTGGAACAGCGTGCGCACCTGCACGCGCAGGCCCCGGGCGCGCACGCGCTCCAGCAGGCCCAGGGCCAGCAGCGAATGCCCGCCCAGCTCGAAGAAGCCGTCCTGCCGTCCCACGCGCTCCACGCCCAGCACCTCGGCCCAGATCTGCGCCAGCGTTTCTTCGAGTTCACCTTGCGGTGCCTCGTATTCCTGCGCACTCGCCATCTCCGGCTCGGGCAGCGCCTTGCGGTCCACCTTGCCGTTGGCCGTCAAAGGCAGGGCATCGAGCACGACGATGGCCGAGGGCACCATGTAGTCGGGCAGCGCCTGGCCCAGGCGCTGCTTGAGCTGGCTTTCCTCCACCGCGTCACGCAGGGAGACATAGGCGATCAGCCTTGCACCCTCCTTGGCCAAAACCACGGCCTCGCGCACCTCGGGCTGGGCCAGCAGTTGCGACTGCACCTCGCCCAGCTCGATGCGGAAGCCCCGGATCTTGACCTGCTGGTCGGCACGACCCAGGTATTCGAGTTCGCCCTGTGCACTCCAGCGCACCAGGTCGCCCGTGCGGTACAGGCGCTCGCCCGTCTCGCTGAACGGGTTGGCAATGAAGCGCTCTGCCGTCAGTCCTTGCCTGTTGAGATAGCCGCGTGCCAAGCCCTCGCCCGCCACATACAACTCTCCCGCCACACCCTGCGGCAGCAGGTTCAGGCTGCCGTCGAGCACGTACAGGCCCAGGTCCGGAATCGCCACGCCCACGGGGCTGCGTCCGCCGTCCAGATCCTTCTGGATGATCTCCCGGTACGTCACATGCACCGTGGTCTCGGTGATGCCGTACATGTTGATGAG


>CP002735.1:2409661-2410699 Delftia sp. Cs1-4, complete genome
CTCATCAACATGTACGGCATCACCGAGACCACGGTGCATGTGACGTACCGGGAGATCACCAAGACTGACC
TTCAAGGAGGACGCAGCCCCGTCGGAGTGGCGATTCCCGACCTGGGCCTGTATGTGCTCGATGGCGGCCT
GGACCTGCTGCCGCAGGGCGTGGCGGGCGAGCTGTTCGTTGCGGGCGCGGGCCTGGCGCGTGGCTACCTG
AACCGGCAAGGCCTGAGCGCGGAGCGCTTCATCGCCAACCCCTTCACGGACGATGGCAGCAGGCTGTACC
GCACAGGCGATCTGGTGCGCTGGAACGCGCAGGGCGAGCTCGAATACCTGGGCCGGGCCGACCAGCAGGT
CAAGATCCGGGGCTTCCGCATCGAGCTGGGCGAAGTGCAGGCCCAGCTGCTGGCGCAGCCGGAGGTGCGT
GAGGCCGTCGTGCTGGCCAGCCAGGGGCCGGGCGGGGCGCGGCTGGTGGCCTATGTCTCGTTGAATTCGC
CAGCCGAAGACGGTTTGCTCAAGGACCGGCTGGGCCATGCGCTGCCCGACTACATGGTGCCCTCGGCCAT
CGTGGTGCTCGATGCCCTGCCGTTGACGGCCAATGGCAAGGTGGACCGCAAGGCCTTGCCTGAACCGGAA
ATGGCAAGTACCCAGCAATACGAGGCGCCGCAGGGCGAACTGGAAGAGGCGCTGGCGCGCATCTGGGCCG
AGGTGCTGGGCGTGGAGCGCGTGGGACGCCATGACAGCTTCTTCGAGCTGGGTGGTCATTCGTTGCTGGC
CCTGAAGCTGCTCGAACGCATGCGCGCCAGGGGGCTGGCGGCCCAGGTGCGCAGCCTGTTCCTTCAGCCC
AGGCTGGCCGACTTTGCGCAGGCCCTTGGTGGCGAACAGGCCGATGCTCGACCCGAGGTGCAGGTGCCGC
CCAACGGCATTCCCGAAGACTGCGCGGCCATCACCCCGGCCATGCTGACCCTGGTGGCGCTGGACGCCCA
GGAGATTGCACGGATCGCCGCAGCCGTGCCCGGTGGTGCAGCCAACATCCAGGACATCT

(Reverse complement):
AGATGTCCTGGATGTTGGCTGCACCACCGGGCACGGCTGCGGCGATCCGTGCAATCTCCTGGGCGTCCAGCGCCACCAGGGTCAGCATGGCCGGGGTGATGGCCGCGCAGTCTTCGGGAATGCCGTTGGGCGGCACCTGCACCTCGGGTCGAGCATCGGCCTGTTCGCCACCAAGGGCCTGCGCAAAGTCGGCCAGCCTGGGCTGAAGGAACAGGCTGCGCACCTGGGCCGCCAGCCCCCTGGCGCGCATGCGTTCGAGCAGCTTCAGGGCCAGCAACGAATGACCACCCAGCTCGAAGAAGCTGTCATGGCGTCCCACGCGCTCCACGCCCAGCACCTCGGCCCAGATGCGCGCCAGCGCCTCTTCCAGTTCGCCCTGCGGCGCCTCGTATTGCTGGGTACTTGCCATTTCCGGTTCAGGCAAGGCCTTGCGGTCCACCTTGCCATTGGCCGTCAACGGCAGGGCATCGAGCACCACGATGGCCGAGGGCACCATGTAGTCGGGCAGCGCATGGCCCAGCCGGTCCTTGAGCAAACCGTCTTCGGCTGGCGAATTCAACGAGACATAGGCCACCAGCCGCGCCCCGCCCGGCCCCTGGCTGGCCAGCACGACGGCCTCACGCACCTCCGGCTGCGCCAGCAGCTGGGCCTGCACTTCGCCCAGCTCGATGCGGAAGCCCCGGATCTTGACCTGCTGGTCGGCCCGGCCCAGGTATTCGAGCTCGCCCTGCGCGTTCCAGCGCACCAGATCGCCTGTGCGGTACAGCCTGCTGCCATCGTCCGTGAAGGGGTTGGCGATGAAGCGCTCCGCGCTCAGGCCTTGCCGGTTCAGGTAGCCACGCGCCAGGCCCGCGCCCGCAACGAACAGCTCGCCCGCCACGCCCTGCGGCAGCAGGTCCAGGCCGCCATCGAGCACATACAGGCCCAGGTCGGGAATCGCCACTCCGACGGGGCTGCGTCCTCCTTGAAGGTCAGTCTTGGTGATCTCCCGGTACGTCACATGCACCGTGGTCTCGGTGATGCCGTACATGTTGATGAG


CLUSTAL O(1.2.4) multiple sequence alignment


CP018101.1:4891000-4892038      AGATGTCCTGGATGTTGGCTGCACCACCGGGCACGGCTGCGGCGATCCGTGCAATCTCCT 60
CP002735.1:2409661-2410699      AGATGTCCTGGATGTTGGCTGCACCACCGGGCACGGCTGCGGCGATCCGTGCAATCTCCT 60
CP022656.1:223076-224129        AGATGTCCTGGATGTTGGCTGCGCCACCGGGCACCGCCTGCGCAATGCGTGCAATCTCAT 60
CP019171.1:4965063-4966107      AGATGTCCTGGATGTTGGCTGCGCCACCGGGCACCGCCTGCGCAATGCGAGCAATCTCCT 60
CP000884.1:5233319-5234363      AGATGTCCTGGATGTTGGCTGCGCCACCGGGCACCGCCTGCGCAATGCGAGCAATCTCCT 60
CP017420.1:7071774-7072827      AGATGTCCTGGATGTTGGCTGCGCCACCGGGCACCGCAGCGGCAATGCGTGCAATCTCAT 60
                                ********************** *********** **    ** ** ** ******** *

CP018101.1:4891000-4892038      GGGCGTCCAGCGCCACCAGGGTCAGCATGGCCGGGGTGATGGCCGTGCAGTCTTCGGGAA 120
CP002735.1:2409661-2410699      GGGCGTCCAGCGCCACCAGGGTCAGCATGGCCGGGGTGATGGCCGCGCAGTCTTCGGGAA 120
CP022656.1:223076-224129        CCTCATCCAGCGCCACCAGGGTCAGCATGTCCGGCGTGATCTCCGTGCAGCCTTCGGGGA 120
CP019171.1:4965063-4966107      CCTCATCCAGCGCCACCAGGGTCAGCATGTCCGGCGTGATCGCCGTGCAGCCTTCGGGGA 120
CP000884.1:5233319-5234363      CCTCATCCAGCGCCACCAGGGTCAGCATGTCCGGCGTGATCGCCGTGCAGCCTTCGGGGA 120
CP017420.1:7071774-7072827      CTTCATCCAGCGCCACCAGGGTCAGCATGTCCGGCGTGATCGCCGTGCAGCCTTCGGGGA 120
                                   * ************************ **** *****  *** **** ******* *

CP018101.1:4891000-4892038      TGCCGTTGGGCGGCACCTGCACCTCGGGTCGAGCATCGGCC------------------- 161
CP002735.1:2409661-2410699      TGCCGTTGGGCGGCACCTGCACCTCGGGTCGAGCATCGGCC------------------- 161
CP022656.1:223076-224129        TGCCGTTGGGCGGCACATCGATCTCGCCGGCCACCTGCGCCCCTGGCTCAC--------- 171
CP019171.1:4965063-4966107      TGCCGTTGGGCGGCACATCGATCTCGCCGGCCACCTGCTCCCCTTGGTCCC--------- 171
CP000884.1:5233319-5234363      TGCCGTTGGGCGGCACATCGATCTCGCCGGCCACCTGCTCCCCTTGGTCCC--------- 171
CP017420.1:7071774-7072827      TGCCATTGGGCGGCACGTCGATCTCGCCGGCCACCTGCGCACCTTGCTCACCCTGCTCAC 180
                                **** *********** * * **** * *   *

CP018101.1:4891000-4892038      -----TGTTCGCCACCAAGGGCCTGCGCAAAGTCGGCCAGCCTGGGCTGAAGGAACAGGC 216
CP002735.1:2409661-2410699      -----TGTTCGCCACCAAGGGCCTGCGCAAAGTCGGCCAGCCTGGGCTGAAGGAACAGGC 216
CP022656.1:223076-224129        CCTGCTGATCCTGCAGCACCGCCTGCGCAAACTCCGCCAACCTCGGGTGCTGGAACAGCG 231
CP019171.1:4965063-4966107      CCTGCTGCGCCTGCAACACTGCCTGCGCAAACTCCGCCAGCCTCGGGTGCTGGAACAGCG 231
CP000884.1:5233319-5234363      CCTGCTGCGCCTGCAACACTGCCTGCGCAAACTCCGCCAGCCTCGGGTGCTGGAACAGCG 231
CP017420.1:7071774-7072827      CCTGCTGCTCCTGCCGCACCGCCTGCGCAAACTCCGCCAGCCTCGGGTGCTGGAACAGCG 240
                                     ** * * *********** ** **** *** ** **  *******

CP018101.1:4891000-4892038      TGCGCACCTGGGCCGCCAGCCCCCTGGCGCGCATGCGTTCGAGCAGCTTCAGGGCCAGCA 276
CP002735.1:2409661-2410699      TGCGCACCTGGGCCGCCAGCCCCCTGGCGCGCATGCGTTCGAGCAGCTTCAGGGCCAGCA 276
CP022656.1:223076-224129        TGCGCACCTGCACGCGCAGGCCCTGGGCGCGCACGCGCTCCAGCAGGCCCAGGGCGAGCA 291
CP019171.1:4965063-4966107      TGCGCACCTGCACGCGCAGGCCCTGGGCGCGCACGCGCTCCAGCAGGCCCAGGGCCAGCA 291
CP000884.1:5233319-5234363      TGCGCACCTGCACGCGCAGGCCCTGGGCGCGCACGCGCTCCAGCAGGCCCAGGGCCAGCA 291
CP017420.1:7071774-7072827      TGCGCACCTGCACGCGCAGGCCCCGGGCGCGCACGCGCTCCAGCAGGCCCAGGGCCAGCA 300
                                ********** * *** *** ******** *** ** *****   ****** ****

CP018101.1:4891000-4892038      ACGAATGACCACCCAGCTCGAAGAAGCTGTCATGGCGTCCCACGCGCTCCACGCCCAGCA 336
CP002735.1:2409661-2410699      ACGAATGACCACCCAGCTCGAAGAAGCTGTCATGGCGTCCCACGCGCTCCACGCCCAGCA 336
CP022656.1:223076-224129        GAGAATGCCCGCCCAGCTCGAAGAAGCCGTCCTGCCGTCCCACGCGCTCCACGCCCAGCA 351
CP019171.1:4965063-4966107      GCGAATGCCCGCCCAGCTCGAAGAAGCCGTCCTGCCGGCCCACGCGATCCACGCCCAGCA 351
CP000884.1:5233319-5234363      GCGAATGCCCGCCCAGCTCGAAGAAGCCGTCCTGCCGGCCCACGCGATCCACGCCCAGCA 351
CP017420.1:7071774-7072827      GCGAATGCCCGCCCAGCTCGAAGAAGCCGTCCTGCCGTCCCACGCGCTCCACGCCCAGCA 360
                                  ***** ** **************** *** ** ** ******** *************

CP018101.1:4891000-4892038      CCTCGGCCCAGATGCGCGCCAGCGCCTCTTCCAGTTCGCCCTGCGGCGCCTCGTATTGCT 396
CP002735.1:2409661-2410699      CCTCGGCCCAGATGCGCGCCAGCGCCTCTTCCAGTTCGCCCTGCGGCGCCTCGTATTGCT 396
CP022656.1:223076-224129        CCTCGGCCCAGATCTTCGCCAGCGTTTCTTCGAGTTCACCTTGCGGTGCCTCGTATTCCT 411
CP019171.1:4965063-4966107      CCTCGGCCCAGATCGTCGCCAGCGTTTCCTCCAACTCGCCCTGCGGTGCCTCGTATTGCT 411
CP000884.1:5233319-5234363      CCTCGGCCCAGATCGTCGCCAGCGTTTCCTCCAACTCGCCCTGCGGTGCCTCGTATTGCT 411
CP017420.1:7071774-7072827      CCTCGGCCCAGATCTGCGCCAGCGTTTCTTCGAGTTCACCTTGCGGTGCCTCGTATTCCT 420
                                ************* ******** ** ** *  ** ** ***** ********** **

CP018101.1:4891000-4892038      GGGTACTTGCCATTTCCGGTTCAGGCAAGGCCTTGCGGTCCACCTTGCCATTGGCCGTCA 456
CP002735.1:2409661-2410699      GGGTACTTGCCATTTCCGGTTCAGGCAAGGCCTTGCGGTCCACCTTGCCATTGGCCGTCA 456
CP022656.1:223076-224129        GCGCACTCGCCATCTCCGGCTCGGGCAGCGCCTTGCGGTCCACCTTGCCGTTGGCAGTCA 471
CP019171.1:4965063-4966107      GGGCGCTGATCATCTCCGGCTCGGGCAGCGCCTTGCGGTCCACCTTGCCGTTGGCTGTCA 471
CP000884.1:5233319-5234363      GGGCGCTGATCATCTCCGGCTCGGGCAGCGCCTTGCGGTCCACCTTGCCGTTGGCTGTCA 471
CP017420.1:7071774-7072827      GCGCACTCGCCATCTCCGGCTCGGGCAGCGCCTTGCGGTCCACCTTGCCGTTGGCCGTCA 480
                                * * ** *** ***** ** **** ******************** ***** ****

CP018101.1:4891000-4892038      ACGGCAGGGCATCGAGCACCACGATGGCCGAAGGCACCATGTAGTCAGGCAGCCCCTGAC 516
CP002735.1:2409661-2410699      ACGGCAGGGCATCGAGCACCACGATGGCCGAGGGCACCATGTAGTCGGGCAGCGCATGGC 516
CP022656.1:223076-224129        GCGGCAGGGCGTCAAGCGCGACGATGGCCGAGGGCACCATGTAGTCGGGCAGCGCCTGGC 531
CP019171.1:4965063-4966107      GCGGCAGGGCTTCGAGCACGACGATGGCCGAGGGCACCATGTAGTCGGGCAGCGCCTGGC 531
CP000884.1:5233319-5234363      GCGGCAGGGCTTCGAGCACGACGATGGCCGAGGGCACCATGTAGTCGGGCAGCGCCTGGC 531
CP017420.1:7071774-7072827      AAGGCAGGGCATCGAGCACGACGATGGCCGAGGGCACCATGTAGTCGGGCAGCGCCTGGC 540
                                  ******** ** *** * *********** ************** ****** * ** *

CP018101.1:4891000-4892038      CCAGCCGCTCCTTGAGCAGCCCATCTTCGACTTCCTCGTTCAGCGAGACATAGGCCACCA 576
CP002735.1:2409661-2410699      CCAGCCGGTCCTTGAGCAAACCGTCTTCGGCTGGCGAATTCAACGAGACATAGGCCACCA 576
CP022656.1:223076-224129        CCAGCCGGTCCTTGAGCCAACCGTCTTCGACCGGCGAATTCAGCGAGACATAGGCCACCA 591
CP019171.1:4965063-4966107      CCAGGCGCTGCTTGAGCTGGCTTTCCTCCACCGCGTCACGCAGGGAGACATAGGCGATCA 591
CP000884.1:5233319-5234363      CCAGGCGCTGCTTGAGCTGGCTTTCCTCCACCGCGTCACGCAGGGAGACATAGGCGATCA 591
CP017420.1:7071774-7072827      CCAGGCGCTGCTTGAGCTGGCTTTCCTCCACCGCGTCACGCAGGGAGACATAGGCGATCA 600
                                **** ** * ******* * ** ** *         ** *********** * **

CP018101.1:4891000-4892038      GGCGCGCCCCGCCCGGCCCCTGGCTGGCCAGCACGACGGCCTCACGCACCTCCGGCTGCG 636
CP002735.1:2409661-2410699      GCCGCGCCCCGCCCGGCCCCTGGCTGGCCAGCACGACGGCCTCACGCACCTCCGGCTGCG 636
CP022656.1:223076-224129        ATCGCGCCCCGCCTGTGCTTTCCTTGGCCAGCACCACGGCCTCGCGCACCTCGGGCTGGG 651
CP019171.1:4965063-4966107      G---------CCTTGCACCCTCCTTGGCCAGCACCACCGCCTCACGCACTTCAGGCTGGG 642
CP000884.1:5233319-5234363      G---------CCTTGCACCCTCCTTGGCCAGCACCACCGCCTCACGCACTTCAGGCTGGG 642
CP017420.1:7071774-7072827      G---------CCTTGCACCCTCCTTGGCCAAAACCACGGCCTCGCGCACCTCGGGCTGGG 651
                                           * * * * ****** ** ** ***** ***** ** ***** *

CP018101.1:4891000-4892038      CCAGCAGCTGGGCCTGCACTTCGCCCAGCTCGATGCGGAAGCCCCGGATCTTGACCTGCT 696
CP002735.1:2409661-2410699      CCAGCAGCTGGGCCTGCACTTCGCCCAGCTCGATGCGGAAGCCCCGGATCTTGACCTGCT 696
CP022656.1:223076-224129        CCAGCAGCTGCGACTGCACCTCGCCCAGCTCGATGCGGAAGCCCCGGATCTTGACCTGCT 711
CP019171.1:4965063-4966107      CCAGCAGCTGCGACTGCACCTCGCCCAGTTCGATGCGGAAGCCCCGGATCTTGACCTGCT 702
CP000884.1:5233319-5234363      CCAGCAGCTGCGACTGCACCTCGCCCAGTTCGATGCGGAAGCCCCGGATCTTGACCTGCT 702
CP017420.1:7071774-7072827      CCAGCAGTTGCGACTGCACCTCGCCCAGCTCGATGCGGAAGCCCCGGATCTTGACCTGCT 711
                                ******* ** * ****** ******** *******************************

CP018101.1:4891000-4892038      GATCGACCCGGCCCAGGTATTCGAGCTCGCCCTGCGCGTTCCAGCGCACCAGGTCGCCTG 756
CP002735.1:2409661-2410699      GGTCGGCCCGGCCCAGGTATTCGAGCTCGCCCTGCGCGTTCCAGCGCACCAGATCGCCTG 756
CP022656.1:223076-224129        GGTCGGCACGGCCCAGGTATTCGAGTTCGCCCTGAGCGTTCCAGCGCACCAGATCGCCCG 771
CP019171.1:4965063-4966107      GGTCGGCACGACCCAGGTATTCGAGTTCGCCCTGTGCACTCCAGCGCACCAGATCGCCCG 762
CP000884.1:5233319-5234363      GGTCGGCACGACCCAGGTATTCGAGTTCGCCCTGTGCACTCCAGCGCACCAGATCGCCCG 762
CP017420.1:7071774-7072827      GGTCGGCACGACCCAGGTATTCGAGTTCGCCCTGTGCACTCCAGCGCACCAGGTCGCCCG 771
                                * *** * ** ************** ******** **  ************* ***** *

CP018101.1:4891000-4892038      TGCGGTACAGCCTGCTGCCATCGTCCGTGAAGGGGTTGGCGATGAAGCGCTCCGCGCTCA 816
CP002735.1:2409661-2410699      TGCGGTACAGCCTGCTGCCATCGTCCGTGAAGGGGTTGGCGATGAAGCGCTCCGCGCTCA 816
CP022656.1:223076-224129        TGCGGTACAGGCGATCGCCTGCCTGTGTAAAGGGATTGGCGATGAAGCGCTCGGCGCTCA 831
CP019171.1:4965063-4966107      TGCGGTACAGGCGATCGCCTGCCTGTGTGAAGGGATTGGCGATAAAGCGCTCGGCGCTCA 822
CP000884.1:5233319-5234363      TGCGGTACAGGCGATCGCCTGCCTGTGTGAAGGGATTGGCGATAAAGCGCTCGGCGCTCA 822
CP017420.1:7071774-7072827      TGCGGTACAGGCGCTCGCCCGTCTCGCTGAACGGGTTGGCAATGAAGCGCTCTGCCGTCA 831
                                ********** * *** * * ** ** ***** ** ******** **  ***

CP018101.1:4891000-4892038      GGCCTTGCCGATTCAGGTAGCCACGCGCCAGGCCCGCGCCCGCAACGAACAGCTCGCCCG 876
CP002735.1:2409661-2410699      GGCCTTGCCGGTTCAGGTAGCCACGCGCCAGGCCCGCGCCCGCAACGAACAGCTCGCCCG 876
CP022656.1:223076-224129        GACCGGCGCGGTTCAGGTAACCACGTGCCAGGCCCTCACCCGCCACATACAACTCTCCCG 891
CP019171.1:4965063-4966107      GGCCAGCGCGATTCAGGTAGCCGCGTGCCAGTCCTTCGCCCGCCACGTACAGCTCCCCGG 882
CP000884.1:5233319-5234363      GGCCAGCGCGATTCAGGTAGCCGCGTGCCAGTCCTTCGCCCGCCACGTACAGCTCCCCGG 882
CP017420.1:7071774-7072827      GTCCTTGCCTGTTGAGATAGCCGCGTGCCAAGCCCTCGCCCGCCACATACAACTCTCCCG 891
                                * ** * ** ** ** ** ** **** **  * ***** ** *** *** ** *

CP018101.1:4891000-4892038      CCACGCCTTGCGGCAACAGGTTCAGACTGCCGTCCAGCACGTACAGGCCCAGGTCCGGAA 936
CP002735.1:2409661-2410699      CCACGCCCTGCGGCAGCAGGTCCAGGCCGCCATCGAGCACATACAGGCCCAGGTCGGGAA 936
CP022656.1:223076-224129        CCACGCCCTGCGGCAGCAGGTTCAGGCTGCCGTCGAGCACGTACAGGCCCAGGTCCGGAA 951
CP019171.1:4965063-4966107      CCACGCCCTGCGGCAGCAGGTTCAGGCTGCCATCGAGCACGTACAGGCCCAGGTCCGGAA 942
CP000884.1:5233319-5234363      CCACGCCCTGCGGCAGCAGGTTCAGGCTGCCATCGAGCACGTACAGGCCCAGGTCCGGAA 942
CP017420.1:7071774-7072827      CCACACCCTGCGGCAGCAGGTTCAGGCTGCCGTCGAGCACGTACAGGCCCAGGTCCGGAA 951
                                **** ** ******* ***** *** * *** ** ***** ************** ****

CP018101.1:4891000-4892038      TGGCAGTTCCCACGGGACTGCGGCCACCGTCCAAATCTGTCTTGGTGATCTCCCGGTACG 996
CP002735.1:2409661-2410699      TCGCCACTCCGACGGGGCTGCGTCCTCCTTGAAGGTCAGTCTTGGTGATCTCCCGGTACG 996
CP022656.1:223076-224129        TCGCCACGCCCACGGGGCTGCGTCCGCCGTCCAGATCCTTCCGGGTGATCTCCCGGTACG 1011
CP019171.1:4965063-4966107      TAGCCACGCCAACGGGGCTGCGGCCACCATCCAAATCCGCCTTGGTGATCTGACGGTACG 1002
CP000884.1:5233319-5234363      TAGCCACGCCAACGGGGCTGCGGCCACCATCCAAATCCGCCTTGGTGATCTGACGGTACG 1002
CP017420.1:7071774-7072827      TCGCCACGCCCACGGGGCTGCGTCCGCCGTCCAGATCCTTCTGGATGATCTCCCGGTACG 1011
                                * ** ** ***** ***** ** ** * *  ** * * ****** *******

CP018101.1:4891000-4892038      TCACATGCACCGTGGTCTCGGTGATGCCGTACATGTTGATGAG 1039
CP002735.1:2409661-2410699      TCACATGCACCGTGGTCTCGGTGATGCCGTACATGTTGATGAG 1039
CP022656.1:223076-224129        TCACATGCACCGTGGTCTCGGTGATGCCGTACATGTTGATGAG 1054
CP019171.1:4965063-4966107      TCACATGCACCGTGGTCTCGGTGATGCCGTACATGTTGATGAG 1045
CP000884.1:5233319-5234363      TCACATGCACCGTGGTCTCGGTGATGCCGTACATGTTGATGAG 1045
CP017420.1:7071774-7072827      TCACATGCACCGTGGTCTCGGTGATGCCGTACATGTTGATGAG 1054
                                *******************************************
