# Supplementary material for: Catalyzing rapid discovery of gold-precipitating bacterial lineages with university students
Source: PeerJ. 2020 Apr 14;8:e8925. doi: 10.7717/peerj.8925 (PMC7164421; doi:10.7717/peerj.8925)
Supplement: Supplemental Information 6 [file peerj-08-8925-s006.docx]

**Supplemental Data S5:** Sanger sequencing results for seventeen environmental samples with sufficient amplified “gold gene” DNA

>Sample_1-3

ATCCAGCGCCACCAGGGTCAGCATGTCCGGCGTGATCGCCGTGCAGCCTTCGGGGATGCCGTTGGGTGGCACGTCGATCT

CGCCGGCCACCTCCTCCCCTTGCTCACCCTGCTGATCCTGCTGCTCCTGCCGTACCGCCTGCGCAAACTCCGCCAGCCTC

GGATGCTGGAACAGCGTGCGCACCTGCACGCGCAGGCCCTGGGCGCGCACACGCTCCAGCAGGCCCAGGGCCAGCAGCGA

ATGCCCACCCAGCTCGAAGAAGCCGTCCTGCCGGCCCACGCGCTCCACGCCCAGCACCTCGGCCCAGATCTGCGCCAGCG

TTTCCTCCAGTTCTCCCTGCGGTGCCTCGTATTGCTGGGCACTGACCATCTCCGGCTCGGGCAGCGCCTTGCGGTCCACC

TTGCCGTTGGCCGTCAAAGGCAGGGCATCGAGCACGACGATGGCCGAGGGCACCATGTAGTCGGGCAGCGCATGGCCCAG

GCGCTGCTTGATCTGGCTTTCCTCCACCGCGTCACGCAGGGAGACATAGGCGATCAGCCTTGCGCCCTCCTTGGCCAAAC

ACCACCGCCTCGCGCACCTCGGGCTGGGCCAGCAGCTGCGACTGCACCTCGCCCAGCTCGATGCGGAAGCCCCGGATCTT

GACCTGCTGGTCGGCACGACCCAGTATTCGAGTTCGCCCTGTGCACTCCAGCGCACCAGGTCGCCCGTGCGGTACAGCGC

TCGCCCGTCTCGCTGAACGGGTTG

>Sample_7-1

TCCAGCGCCACCAGGGTCAGCATGTCCGGCGTGATCGCCATGCAGCCTTCGGGGATGCCGTTGGGCGGCACATCGATCTC

GCCGGCCACCTGCGCCCCTTGCTCACCCTGCTGATCCTGCCGCACCGCCTGCACAAACTCTGCCAACCTCGGGTGCTGGA

ACAGCTTGAGCACCTGGACGCGCAGGCCCCGGGCGCGCAGGCGCTCCACCAGGCCCAGGGCGAGCAGGGAATGCCCGCCC

AGCTCGAACAAGCCGTCCTGCCGGCCCACGCGCTCCACGCCCAGCACCTCGGCCCACATCTGCGCCATCGTTTCCTCCAG

CTCTCCCTGCGGTGCCTCGTATTGCTGGGAGCTGAGCATCTCCGGCTCGGGCAGCGCCTTGCGGGCCATCTTGCCGTTGG

TGGTCAAAGGCAGGGCGTCGAGGACGACGATGGCCAAGGGCACCATGTAGTCCGGGAGCACCTGGACCAGTCGTCCCTTG

AGCTGACCCTCTGCGATTTCTGCATTCAGCGACACATAGGCGATCAATCTGATAACGCCCGCGCCCTCCTTGGCCAGCAC

CACTGCCTCGCGCACCTTCGGGCTGGGCCAGCAGTTGCGACTGCACCTCGCCCAGCTCGATGCGGAAGCCCCGGATCTTG

ACCTGCTGGTCGGCACGGCCCAGTATTCGAGTTCGCCCTGAGCGTTCCAGCGCACCAAGTCGCCCGTGCGGTACA

>Sample_7-2

TCCAGCGCCACCAGGGTCAGCATGTCCGGCGTGATCACCGTGCAGCCTTCGGGGATGCCGTTGGGTGGCACATCGATCTC

GCCGGCCACCTGCTCACCTTGCTGATCCTGCTGTTCCTGCTGTTCCTCCTGTACAAACTGCGCCAACTTCGGATGCTGGA

AAAGCTGGAACACCTGGCCCCCCTGGACCTGGGCGCGCAGGGGCTCCACCAGGTCCAGGGGGAGCAGGGAAAGCCCCCAA

TGCTCGAACAAGTCGTCCAGCCCTCCCTGGCGCTCCACGCCCACAACGTCCGCCAAGATCTTCCACATCTTTTCTTCCAG

TTCTTCTTGCTGTGCCTGGTATTCCTGCTATTGCTGCATCTTCGGCTTGTGCAGCTCGTTGAGCTCCTTGTTGCCCATGG

TGGTCTTAGGCATGGCTTCCAGCACAACGATGGCCAAAAGCACCGAGTAGTCCGTGAACGCCTGGACCACACGTCCCTTG

AGCTGATTGATCTGGATTTCCGCATTCACGTACACATAGGAGATATATCTGATCACGCCTGCCCCCTCCTTGGCCAGCAC

CACCGCCTCGCGCACCTCGGGCTGGGCCAGCAGCTGCGACTGCACCTCGCCCAGCTCGATGCGGAAGCCCCGGATCTTGA

CCTGCTGGTCGGCACGGCCCAGTATTCGAGTTCGCCCTGAGCA

>Sample_9-1

TCCAGCGCCACCAGGGTCAGCATGTCCGGCGTGATCGCCGTGCAGCCTTCGGCGATGCCGTTGGGCGGCACGTCGATCTC

GCCGGCTACCCGCTCCCCTTGGTCCCCCTGCTGCTCCTGCAGCACCGCCTGCGCAAACTCCGCCAGCCTCGGGTGCTGGA

ACAGCGTGCGCATCTGCACGCGCAGGCCCTGGGCGCGCACGCGCTCCAGCAGGCCCAGGGCGAGCAGCGAATGCCCGCCC

AGCTCGAAGAAGCCGTCCTGCCGGCCCACGCGCTCCACGCCCAGCACCTCGGCCCAGATCTGCGCCAGCGTTTCTTCCAG

TTCACCTTGCGGTGCCTCGTATTCCTGCGCACTCACCATCTCCGGCTCGGGCAGCGCCTTGCGGTCCACCTTGCCGTTGG

CCGTCAAAGGCAGGGCATCGAGCACGACGATGGCCGAGGGCACCATGTAGTCGGGCAGTACCTGGCCCAGGCGCTGCTTG

AGCTGGCTTTCCTCCACCGCGTCACGCAGGGAGACATAGGCGATCAGCCTTGCACCCTCCTTGGCCAGCACCACGGCCTC

GCGCACCTCGGGCTGGGCCAGC

>Sample_12-2

GCATGCGTGCATCTCATCTTCATCCAGCGCCACCAGGGTCAGCATGTCCGGCGTGATCGCCGTGCAGCCTTCGGGGATGC

CATTGGGCGGCACGTCGATCTCGCCGGCCACCTGCGCCCCTTGCTCACCCTGCTCACCCTGCTGCTCCTGCCGCACCGCC

TGCGCAAACTCCGCCAGCCTCGGGTGCTGGAACAGCGTGCGCACCTGCACGCGCAGGCCCTGGGCGCGCACGCGCTCCAG

CAGGCCCAGGGCGAGCAGCGAATGCCCGCCCAGTTCAAAGAAGCCGTCCTGCCGGCCCACGCGATCCACGCCCAGCACGT

CCGCCCAGATCTGCGCCAGCGTTTCTTCCAACTCGCCCTGTGGTGCCTCGTATTGCTGGGCGCTGACCATCTCCGGCTCG

GGCAGCGCCTTGCGGTCCACCTTGCCGTTGGCCGTCAGCGGCAGGGCGTCAAGCACGACGATGGCCGAGGGCACCATGTA

GTCGGGCAGCGACTGGCCCAGCCGCTGCTTGAGCTGGCTTTCCTCCACCGCGTCACGCAGGGAGACATAGGCGATCAGCC

TTGCGCCCTCCTTGGCCAAAACCACGGCCCCGCGCACCTCGGGCTGGGCCAGCAGCTGCGACTGCACCTCGCCCAGCTCG

ATGCGGAAGCCCCGGATCTTGACCTGCTGGTCGGCACGGCCCAGTATTCAAGTTCGCCCTGGGCACTCCAGCGCACCAGT

CGCCCGTGCGGTACAGGCGCTCGCCCGTCTCGCTGACGGG

>Sample_15-1

ATCCAGCGCCACCAGGGTCAGCATGTCCGGCGTGATCGCCGTGCAGCCTTCGGGGATGCCGTTGGGTGGCACATCGATCT

CGCCGGCTACCCGCTCCCCCCGCTCACCCTGCTGCTCCTGCTGTTCCTCCAGTACCGCCTGCGCAAACTCCGCCAGCCTC

GGATGCTGGAACAGCGTGCGCACCTGCACGCGCAGGCCCTGGGCGCGCACGCGCTCCAGCAGGCCCAGGGCGAGCAGCGA

ATGCCCGCCCAGTTCAAAGAAGCCGTCCTGCCGGCCCACGCGATCCACGCCCAGCACGTCCGCCCAGATCTGCGCCAGCG

TTTCTTCCAACTCGCCCTGTGGTGCCTCGTATTGCTGGGCGCTGACCATCTCCGGCTCGGGCAGCGCCTTGCGGTCCACC

TTGCCGTTGGCCGTCAGCGGCAGGGCATCGAGCACGACGATGGCCGAGGGCACCATGTAGTCGGGCAGCGCCTGGCCCAG

GCGCTGCTTGATCTGGCTTTCCTCCACCGCGTCACGCAGGGAGACATAGGCGATCAGCCTTGCGCCCTCCTTGGCCAGCA

CCACCGCCTCGCGCACCTCGGGCTGGGCCAGCAGCTGCGACTGCACCTCGCGCAGCTCGATACGGAAGCCCCGGATCTTG

ACCTGCTGGTCG

>Sample_15-2

CCAGGGTCAGCATGTCCGGCGTGATCGCCGTGCAGCCTTCGGGGATGCCGTTGGGTGGCACATCGATTTCGCCGGCTACC

CGCTCCCCTTGCTCACCCTGCTGCTCCTGCCGCACCGCCTGCGCAAACTCCGCCAGCCTCGGATGCTGGAACAGCGTGCG

CACCTGCACGCGCAGGCCCTGGGCGCGTACGCGCTCCAGCAGGCCCAGGGCGAGCAGCGAATGCCCGCCCAGCTCGAAGA

AGCCGTCCTGCCGGCCCACGCGCTCCACGCCCAGCACCTCGGCCCAGATCTTCGCCAGCGTTTCTTCGAGTTCACCTTGC

GGTGCCTCGTATTCCTGCGCACTCGCCATCTCCGGCTCGGGCAGCGCCTTGCGGTCCACCTTGCCATTGGCTGTCAGCGG

CAGGGCTTCGAGCACGACGATGGCCGAGGGCACCATGTAGTCGGGCAGCGCCTGGCCCAGCCGCTGCTTGAGCTGGCTTT

CCTCCACCGCGTCACGCACGGAGACATAGGCGATCAGCCTTGCACCATCCTTGGCCAAAACCACGGCCTCGCGCACCTCG

GTCTGGGCCAGCAGCTGCGACTGCACCTCGCCCAGCTCGATGCGGAAGCCCCGGATCTTGACCTGCTGG

>Sample_17-3

GGCATGCGTGCATCTCATCTTCATCCAGCGCCACCAGGGTCAGCATGTCCGGCGTGATCGCCGTGCAGCCTTCGGGGATG

CCATTGGGCGGCACGTCGATCTCGCCGGCCACCTGCGCCCCTTGCTCACCCTGCTCACCCTGCTGCTCCTGCCGCACCGC

CTGCGCAAACTCCGCCAGCCTCGGGTGCTGGAACAGCGTGCGCACCTGCACGCGCAGGCCCTGGGCGCGCACGCGCTCCA

GCAGGCCCAGGGCGAGCAGCGAATGCCCGCCCAGTTCAAAGAAGCCGTCCTGCCGGCCCACGCGATCCACGCCCAGCACG

TCCGCCCAGATCTGCGCCAGCGTTTCTTCCAACTCGCCCTGTGGTGCCTCGTATTGCTGGGCGCTGACCATCTCCGGCTC

GGGCAGCGCCTTGCGGTCCACCTTGCCGTTGGCCGTCAGCGGCAGGGCGTCAAGCACGACGATGGCCGAGGGCACCATGT

AGTCGGGCAGCGACTGGCCCAGCCGCTGCTTGAGCTGGCTTTCCTCCACCGCGTCACGCAGGGAGACATAGGCGATCAGC

CTTGCGCCCTCCTTGGCCAAAACCACGGCCCCGCGCACCTCGGGCTGGGCCAGCAGCTGCGACTGCACCTCGCCCAGCTC

GATGCGGAAGCCCCGGATCTTGACCTGCTGGTCGGCACGGCCCACGTATTCAAGTTCGCCCTGGGCACTCCAGCGCACCA

GTCGCCCGTGCGGTACAGGCGCTCGCCCGTCTCGCTGAACGGGTTGG

>Sample_18-1

TAGTCCGTGCGTGATCGCCGTGCAGCCTTCGGGGATGCCGTTGGGCGGCACATCGATCTCGCCGGCCACCTGCTCCCCTT

GCTCACCCTGCTGCTCCTGCTGCACCGCCTGCGCAAACTCCGCCAGCCTCGGGTGCTGGAACAGCGTGCGCACCTGCACG

CGCAGGCCCTGGGCGCGCACGCGCTCCAGCAGGCCCAGGGCGAGCAGCGAATGCCCGCCCAGCTCGAAGAAGCCGTCCTG

CCGGCCCACGCGCTCCACGCCCAGCACCTCGGCCCAGATCTGCGCCAGCGTTTCCTCCAGTTCTCCCTGCGGTGCCTCGT

ATTGCTGGGCGCTCACCATCTCCGGCTCGGGCAGCGCCTTGCGGTCCACCTTGCCGTTGGCCGTCAAAGGCAGGGCATCG

AGCACGACGATGGCCGAGGGCACCATGTAGTCGGGCAGTACCTGGCCCAGCCGGTCCTTGAGCTGACTGTCTTCGATTTC

TGCATTCAGCGACACATAGGCGATAAGTCTGACACCGCCTGCGCCCTCCTTGGCCAGCACCACCGCCTCACGCACCTCGG

GCTGGGCCAGCAGCTGCGACTGCACCTCGCCCAGCTCGATGCGGAAGCCCCGGATCTTGACCTGCTGGTCGG

>Sample_23-2

ATGCGCGCATCTCCTCCTCATCCAGCGCCACCAGGGTCAGCATGTCCGGCGTGATCGCCGTGCAGCCTTCGGGGATGCCG

TTGGGTGGCACATCGATCTCGCCGGCCACCTGCTCACCCTGCTGCTCCTGCTGTTCCTCCAATACCGCCTGCGCAAACTC

CGCCAGCCTCGGATGCTGGAACAGCGTGCGCACCTGCACGCGCAGGCCCTGGGCGCGCACGCGCTCCAGCAGGCCCAGGG

CCAGCAGCGAATGCCCGCCCAGATCGAAGAAGCCGTCCTGCCGGCCCACGCGCTCCACGCCCAGCACCTCGGCCCAGATC

TGCGCCAGCGTTTCCTCCAACTCGCCCTGCGGTGCCTCATATTGCTGGGCGCTGACCATCTCCGGCTCGGGCAGCGCCTT

GCGGTCCACCTTGCCGTTGGCGGTCAAAGGCAGGGCATCGAGCACGACGATGGCCGAGGGCACCATGTAGTCGGGCAGTA

TCTCGCCCAGCCGGTCCTTGAGCAGACCCTCTGCGATTTCTGCATTCAGCGACACATAGGCGATCAGTCTGGCACCGCCC

GCCCCCTCCTTGGCCAGCACCACCGCCTCACGCACTTCAAGCTGGGCCAGCAGCTGCGACTGCACCTCGCCCAGCTCGAT

GCGGAAGCCCCGGATCTTGACCTGCTGGTCGGCACGGTCCAGTATTCCAGTTCGCCCTGAGCGTTCCAGCGCACCAGATC

GCCCGTGCGGTACAGACGCTCGCC

>Sample_24-1

CCAGGGTCAGCATGTCCGGCGTGATCGCCGTGCAGCCTTCGGGGATGCCGTTGGGTGGCACATCGATCTCGCCGGCCACC

TGCTCACCCTGCTGCTCCTGCTGTTCCTCCAATACCGCCTGCGCAAACTCCGCCAGCCTCGGATGCTGGAACAGCGTGCG

CACCTGCACGCGCAGGCCCTGGGCGCGCACGCGCTCCAGCAGGCCCAGGGCCAGCAGCGAATGCCCGCCCAGATCGAAGA

AGCCGTCCTGCCGGCCCACGCGCTCCACGCCCAGCACCTCGGCCCAGATCTGCGCCAGCGTTTCCTCCAACTCGCCCTGC

GGTGCCTCATATTGCTGGGCGCTGACCATCTCCGGCTCGGGCAGCGCCTTGCGGTCCACCTTGCCGTTGGTGGTCAAAGG

CAGGGCATCGAGCACGACGATGGCCGAGAGCACCATGTAGTCGGGCAGTATCTCGCCCAGCCGGTCCTTGAGCAGACCCT

CTGCGATTTCTGCATTCAGCGACACATAGGCGATCAGTCTGGCACCGCCCGCCCCCTCCTTGGCCAGCACCACCGCCTCA

CGCACTTCAGGCTGGGCCAGCAGTTGCGACTGCACCTCGCCCAGCTCGATGCGGAAGCCCCGGATCTTGACCTGCTGGTC

GGCACGGCCCAGGTATTCGAGTTCGCCCTGAGCGTTCCAGCGCACCAGATC

>Sample_25-1

TCCTCATCCAGCGCCACCAGGGTCAGCATGTCCGGCGTGATCGCCGTGCAGCCTTCGGCGATGCCGTTGGGCGGCACGTC

GATCTCGCCGGCTACCCGCTCCCCTTGGTCCCCCTGCTGCTCCTGCAACACCGCCTGCGCAAACTCCGCCAGCCTCGGGT

GCTGGAACAGCGTGCGCATCTGCACGCGCAGGCCCTGGGCGCGCACGCGCTCCAGCAGGCCCAGGGCGAGCAGCGAATGC

CCGCCCAGCTCGAAGAAGCCGTCCTGCCGGCCCACGCGCTCCACGCCCAGCACCTCGGCCCAGATCTGCGCCAGCGTTTC

TTCCAGTTCACCTTGCGGTGCCTCGTATTCCTGCGCACTCACCATCTCCGGCTCGGGCAGCGCCTTGCGGTCCACCTTGC

CGTTGGCCGTCAAAGGCAGGGCATCGAGCACGACGATGGCCGAGGGCACCATGTAGTCGGGCAGTACCTGGCCCAGGCGC

TGCTTGAGCTGACTTTCCTCCACCGCGTCACGCAGGGAGACATAGGCGATCAGCCTTGCACCCTCCTTGGCCAGCATCAC

GGCCTCGCGCACCTCGGGCTGGGCCAGC

>Sample_25-2

AGCGCCACCAGGGTCAGCATGTCCGGCGTGATCGCCGTGCAGCCTTCGGGGATGCCGTTGGGTGGCACATCGATCTCGCC

GGCCACCTGCTCCCCTTGCTCACCCTGCTGCTCCTGCTGCTCCTGCCGCACCGCCTGCGCAAACTCCGCCAGCCTCGGAT

GCTGGAACAGCGTGCGCACCTGGACGCGCAGGCCCTGGGCGCGCACACGCTCCAGCAGGACCAGGGCCAGCAGCGAATGC

CCGCCCAACTCGAAGAAGCCGTCCTGCCGGCCCACGCGCTCCACGCCCAGCACCTCGGCCCAGATCTGCGCCAGCGTTTC

TTCTAGTTCTGCCTGCGGTGCCTCGTATTGCTGCGCACTCAGCATCTCCGGCTCGGGCAGCGCCTTGCGGTCCACCTTGC

CGTTGGCCGTCAAAGGCAGGGCATCGAGCACGACGATGGCCGAGGGCACCATGTAGTCGGGCAGCGCCTGGCCCATGCGC

TGCTTGATCTGGCTTTCCTCCACCGCGTCACGCAAGGAGACATAGGCGATCAGCCTTGCGCCCTCCTTGGCCAAAACCAC

GGCCTCGCGCACCTCGAGCTGGGCCAGCAGCTGCGACTGCACCTCGCCCAGCTCGATGCGGAAGCCCCGGATCTTGACCT

GCTGGTCGGCAC

>Sample_26-1

TCCAGCGCCACCAGGGTCAGCATGTCCGGCGTGATCGCCGTGCAGCCTTCGGGGATGCCGTTGGGCGGCACATCGATCTC

GCCGGCCACCTGCACCCCTTGCTCACCCTGCTGCTCCTGCTGTTCCTCCAGTACCGCCTGCGCAAACTCCGCCAGCCTCG

GATGCTGGAACAGCGTGCGCACCTGCACGCGCAGGCCCTGGGCGCACACGCGCTCCAGCAGGCCCAGGGCCAGCAGCGAA

TGCCCGCCCAGTTCAAAGAAGCCGTCCTGCCGGCCCACGCGATCCACGCCCAGCACGTCCGCCCAGATCTGCGCCAGCGT

TTCTTCCAACTCGCCCTGTGGTGCCTCGTATTGCTGGGCGCTGACCATCTCCGGCTCGGGCAGCGCCTTGCGGTCCACCT

TGCCGTTGGCAGTCAAAGGCAGGGCATCGAGCACGACGATGGCCGAGGGCACCATGTAGTCGGGCAGCACATGGCCCAGG

CGCTGCTTGATCTGACTTTCCTCCATCGCGTCATTCAGGGAGACATAGGCGATCAGTCTTGCACCCTCCTTGG

>Sample_30-2

CATGCGTGCATCTCCTCCTCATCCAGCGCCACCAGGGTCAGCATGTCCGGCGTGATCGCCGTGCAGCCTTCGGGGATGCC

GTTGGGCGGCACATCGATCTCGCCGGCCACCTGCTCCCCTTGCTCACCCTGCTGCTCCTGCCGCACCGCCTGCGCAAACT

CCGCCAGCCCCGGATGCTGGAACAGCGTGCGCACCTGCACGCGCAGGCCCTGGGCGCGTACGCGCTCCAGCAGGCCCAGG

GCGAGCAGCGAATGCCCGCCCAGCTCGAAGAAGCCGTCCTGCCGGCCCACGCGCTCCACGCCCAGCACCTCGGCCCAGAT

CTTCGCCAGCGTTTCTTCGAGTTCACCTTGCGGTGCCTCGTATTCCTGCGCACTCGCCATCTCAGGCTCGGGCAGCGCCT

TGCGGTCCACCTTGCCGTTGGCTGTCAGCGGCAGGGCTTCGAGCACGACGATGGCCGAGGGCACCATGTAGTCGGGCAGC

GCCTGGGCCAGCCGCTGCTTGAGCTGGCTTTCCTCCACCGCGTCACGCAGGGAGACATAGGCGATCAGCCTTGCACCATC

CTTGGCCAAAACCACGGCCTCGCGCACCTCGGGCTGGGCCAGCAGCTGCGACTGCACCTCGCCCAGTTCGATGCGGAAGC

CCCGGATCTTGACCTGCTGGTCGGCACGGCCCACGTATTCGAGTTCGCCCTGAGTGTTCCAGCGCACCAGATCGCCCGTG

CGGTACAGGCGCTCGCCCGTCTCGCTGAACGGG

>Sample_30-3

AGCGCCACCAGGGTCAGCATGTCCGGCGTGATCACCGTGCAGCCTTCGGGGATGCCGTTGGGTGGCACATCGATCTCGCC

GGCCACCTGCTCACCCTGCTGCTCCTGCTGTTCCTCCAGTACCGCCTGCGCAAACTCCGCCAGCCTCGGATGCTGGAACA

GCGTGCGCACCTGCACGCGCAGGCCCTGGGCGCGCACGCGCTCCAGCAGGCCCAGGGCGAGCAGCGAATGCCCGCCCAGC

TCGAAGAAGCCGTCCTGCCGTCCCACGCGCTCCACGCCCAGAACGTCCGCCCAGATCTTCGCCAGCGTTTCTTCGAGTTC

ACCTTGCGGTGCCTCGTATTCCTGCGCACTCGCCATCTCCGGCTCGGGCAGCGCCTTGCGGTCCACCTTGCCGTTGGCTG

TCAGCGGCAGGGCGTCGAGCACGACGATGGCCGAAGGCATCATGTAGTCGGGCAGCGCATGGCCCAGCCGGTCCTTGAGC

AAACCGTCTTCGGCTGGCGAATTCAGCGAGACATAGGCCACCAATCGCGCCCCGCCTGCGCTTTGCTTGGCCAGCACCAC

CGCCTCACGCACTTCAGGCTGGGCCAGCAGTTGCGACTGCACCTCGCCCAGCTCGATGCGGAAGCCCCGGATCTTCACCT

GCTGGTCGGCACGGCCCACGTATTCGAGTTCGCCCTGAGCGTTCCAGCGCACCAGATCGCCTGTGCGGTACAGGCGCTCG

CCG

>Sample_33-2

TCCAGCGCCACCAGGGTCAGCATGTCCGGCGTGATCGCCGTGCAGCCTTCGGGGATGCCGTTGGGCGGCACGTCGATCTC

GCCGGCCACCTCCTCCCCTTGCTCACCCTGCTGATCCTGCTGCTCCTGCCGTACCGCCTGCGCAAACTCCGCCAGCCTCG

GATGCTGGAACAGCGTGCGCACCTGCACGCGCAGGCCCCGGGCGCGCACGCGCTCCAGCAGGCCCAGGGCCAGCAGCGAA

TGCCCGCCCAGCTCGAAGAAGCCGTCCTGCCGGCCCACGCGCTCCACGCCCAGCACCTCGGCCCAGATCTGCGCCAGCGT

TTCTTCCAGTTCTCCTTGCGGTGCCTCGTATTGCTGGGCACTCACCATCTCCGGCTCGGGCAGCGCCTTGCGGTCCACCT

TGCCGTTGGCCGTCAAAGGCAGGGCATCGAGCACGACGATGGCCGAGGGCACCATGTAGTCGGGCAGCGCCTGGCCCAGG

CGCTGCTTGATCTGGCTTTCCTCCACCGCGTCACGCAGGGAGACATAGGCGATCAGCCTTGCACCCTCCTTGGCCAAAAC

CACCGCCTCGCGCACCTCGGGCTGGGCCAGCAGCTGCGACTGCACCTCGCCCAGCTCGATGCGGAAGCCCCGGATCTTGA

CCTGCTGGTCGGCACGACCCAGTATTCGAGTTCGCCCTGTGCACTCCAGCGCACCAGTCGCCCGTGCGGTACAGGCGCTC

GCCCGTCTCGCTGACGGGTTGGC
