# Supplementary material for: Catalyzing rapid discovery of gold-precipitating bacterial lineages with university students
Source: PeerJ. 2020 Apr 14;8:e8925. doi: 10.7717/peerj.8925 (PMC7164421; doi:10.7717/peerj.8925)
Supplement: Supplemental Information 7 [file peerj-08-8925-s007.docx]

**Supplemental Data S6:** Alignment of *D. tsuruhatensis* strain CM13 and environmental samples

Sample_12-2      -------------------------------------------GCATGCGTGCATCTCAT 17
Sample_17-3      ------------------------------------------GGCATGCGTGCATCTCAT 18
Sample_15-1      ------------------------------------------------------------ 0
Sample_26-1      ------------------------------------------------------------ 0
Sample_1-3       ------------------------------------------------------------ 0
Sample_25-2      ------------------------------------------------------------ 0
Sample_33-2      ------------------------------------------------------------ 0
Sample_15-2      ------------------------------------------------------------ 0
Sample_30-2      --------------------------------------------CATGCGTGCATCTCCT 16
Delftia          AGATGTCCTGGATGTTGGCTGCGCCACCGGGCACCGCAGCGGCAATGCGTGCAATCTCAT 60
Sample_9-1       ------------------------------------------------------------ 0
Sample_25-1      -----------------------------------------------------------T 1
                                                                            

Sample_12-2      CTTCATCCAGCGCCACCAGGGTCAGCATGTCCGGCGTGATCGCCGTGCAGCCTTCGGGGA 77
Sample_17-3      CTTCATCCAGCGCCACCAGGGTCAGCATGTCCGGCGTGATCGCCGTGCAGCCTTCGGGGA 78
Sample_15-1      ----ATCCAGCGCCACCAGGGTCAGCATGTCCGGCGTGATCGCCGTGCAGCCTTCGGGGA 56
Sample_26-1      -----TCCAGCGCCACCAGGGTCAGCATGTCCGGCGTGATCGCCGTGCAGCCTTCGGGGA 55
Sample_1-3       ----ATCCAGCGCCACCAGGGTCAGCATGTCCGGCGTGATCGCCGTGCAGCCTTCGGGGA 56
Sample_25-2      --------AGCGCCACCAGGGTCAGCATGTCCGGCGTGATCGCCGTGCAGCCTTCGGGGA 52
Sample_33-2      -----TCCAGCGCCACCAGGGTCAGCATGTCCGGCGTGATCGCCGTGCAGCCTTCGGGGA 55
Sample_15-2      ---------------CCAGGGTCAGCATGTCCGGCGTGATCGCCGTGCAGCCTTCGGGGA 45
Sample_30-2      CCTCATCCAGCGCCACCAGGGTCAGCATGTCCGGCGTGATCGCCGTGCAGCCTTCGGGGA 76
Delftia          CTTCATCCAGCGCCACCAGGGTCAGCATGTCCGGCGTGATCGCCGTGCAGCCTTCGGGGA 120
Sample_9-1       -----TCCAGCGCCACCAGGGTCAGCATGTCCGGCGTGATCGCCGTGCAGCCTTCGGCGA 55
Sample_25-1      CCTCATCCAGCGCCACCAGGGTCAGCATGTCCGGCGTGATCGCCGTGCAGCCTTCGGCGA 61
                                ****************************************** **

Sample_12-2      TGCCATTGGGCGGCACGTCGATCTCGCCGGCCACCTGCGCCCCTTGCTCACCCTGCTCAC 137
Sample_17-3      TGCCATTGGGCGGCACGTCGATCTCGCCGGCCACCTGCGCCCCTTGCTCACCCTGCTCAC 138
Sample_15-1      TGCCGTTGGGTGGCACATCGATCTCGCCGGCTACCCGCTCCCCCCGCTCACCCTGCTGCT 116
Sample_26-1      TGCCGTTGGGCGGCACATCGATCTCGCCGGCCACCTGCACCCCTTGCTCACCCTGCTGCT 115
Sample_1-3       TGCCGTTGGGTGGCACGTCGATCTCGCCGGCCACCTCCTCCCCTTGCTCACCCTGCTGAT 116
Sample_25-2      TGCCGTTGGGTGGCACATCGATCTCGCCGGCCACCTGCTCCCCTTGCTCACCCTGCTGCT 112
Sample_33-2      TGCCGTTGGGCGGCACGTCGATCTCGCCGGCCACCTCCTCCCCTTGCTCACCCTGCTGAT 115
Sample_15-2      TGCCGTTGGGTGGCACATCGATTTCGCCGGCTACCCGCTCCCCTTG---------CTCAC 96
Sample_30-2      TGCCGTTGGGCGGCACATCGATCTCGCCGGCCACCTGCTCCCCTTG---------CTCAC 127
Delftia          TGCCATTGGGCGGCACGTCGATCTCGCCGGCCACCTGCGCACCTTGCTCACCCTGCTCAC 180
Sample_9-1       TGCCGTTGGGCGGCACGTCGATCTCGCCGGCTACCCGCTCCCCTTGG---------TCCC 106
Sample_25-1      TGCCGTTGGGCGGCACGTCGATCTCGCCGGCTACCCGCTCCCCTTGG---------TCCC 112
                 **** ***** ***** ***** ******** ***  * * ** * *

Sample_12-2      CCTGCTGCTCCTGCCGCACCGCCTGCGCAAACTCCGCCAGCCTCGGGTGCTGGAACAGCG 197
Sample_17-3      CCTGCTGCTCCTGCCGCACCGCCTGCGCAAACTCCGCCAGCCTCGGGTGCTGGAACAGCG 198
Sample_15-1      CCTGCTGTTCCTCCAGTACCGCCTGCGCAAACTCCGCCAGCCTCGGATGCTGGAACAGCG 176
Sample_26-1      CCTGCTGTTCCTCCAGTACCGCCTGCGCAAACTCCGCCAGCCTCGGATGCTGGAACAGCG 175
Sample_1-3       CCTGCTGCTCCTGCCGTACCGCCTGCGCAAACTCCGCCAGCCTCGGATGCTGGAACAGCG 176
Sample_25-2      CCTGCTGCTCCTGCCGCACCGCCTGCGCAAACTCCGCCAGCCTCGGATGCTGGAACAGCG 172
Sample_33-2      CCTGCTGCTCCTGCCGTACCGCCTGCGCAAACTCCGCCAGCCTCGGATGCTGGAACAGCG 175
Sample_15-2      CCTGCTGCTCCTGCCGCACCGCCTGCGCAAACTCCGCCAGCCTCGGATGCTGGAACAGCG 156
Sample_30-2      CCTGCTGCTCCTGCCGCACCGCCTGCGCAAACTCCGCCAGCCCCGGATGCTGGAACAGCG 187
Delftia          CCTGCTGCTCCTGCCGCACCGCCTGCGCAAACTCCGCCAGCCTCGGGTGCTGGAACAGCG 240
Sample_9-1       CCTGCTGCTCCTGCAGCACCGCCTGCGCAAACTCCGCCAGCCTCGGGTGCTGGAACAGCG 166
Sample_25-1      CCTGCTGCTCCTGCAACACCGCCTGCGCAAACTCCGCCAGCCTCGGGTGCTGGAACAGCG 172
                 ******* **** * ************************* *** *************

Sample_12-2      TGCGCACCTGCACGCGCAGGCCCTGGGCGCGCACGCGCTCCAGCAGGCCCAGGGCGAGCA 257
Sample_17-3      TGCGCACCTGCACGCGCAGGCCCTGGGCGCGCACGCGCTCCAGCAGGCCCAGGGCGAGCA 258
Sample_15-1      TGCGCACCTGCACGCGCAGGCCCTGGGCGCGCACGCGCTCCAGCAGGCCCAGGGCGAGCA 236
Sample_26-1      TGCGCACCTGCACGCGCAGGCCCTGGGCGCACACGCGCTCCAGCAGGCCCAGGGCCAGCA 235
Sample_1-3       TGCGCACCTGCACGCGCAGGCCCTGGGCGCGCACACGCTCCAGCAGGCCCAGGGCCAGCA 236
Sample_25-2      TGCGCACCTGGACGCGCAGGCCCTGGGCGCGCACACGCTCCAGCAGGACCAGGGCCAGCA 232
Sample_33-2      TGCGCACCTGCACGCGCAGGCCCCGGGCGCGCACGCGCTCCAGCAGGCCCAGGGCCAGCA 235
Sample_15-2      TGCGCACCTGCACGCGCAGGCCCTGGGCGCGTACGCGCTCCAGCAGGCCCAGGGCGAGCA 216
Sample_30-2      TGCGCACCTGCACGCGCAGGCCCTGGGCGCGTACGCGCTCCAGCAGGCCCAGGGCGAGCA 247
Delftia          TGCGCACCTGCACGCGCAGGCCCCGGGCGCGCACGCGCTCCAGCAGGCCCAGGGCCAGCA 300
Sample_9-1       TGCGCATCTGCACGCGCAGGCCCTGGGCGCGCACGCGCTCCAGCAGGCCCAGGGCGAGCA 226
Sample_25-1      TGCGCATCTGCACGCGCAGGCCCTGGGCGCGCACGCGCTCCAGCAGGCCCAGGGCGAGCA 232
                 ****** *** ************ ******  ** ************ ******* ****

Sample_12-2      GCGAATGCCCGCCCAGTTCAAAGAAGCCGTCCTGCCGGCCCACGCGATCCACGCCCAGCA 317
Sample_17-3      GCGAATGCCCGCCCAGTTCAAAGAAGCCGTCCTGCCGGCCCACGCGATCCACGCCCAGCA 318
Sample_15-1      GCGAATGCCCGCCCAGTTCAAAGAAGCCGTCCTGCCGGCCCACGCGATCCACGCCCAGCA 296
Sample_26-1      GCGAATGCCCGCCCAGTTCAAAGAAGCCGTCCTGCCGGCCCACGCGATCCACGCCCAGCA 295
Sample_1-3       GCGAATGCCCACCCAGCTCGAAGAAGCCGTCCTGCCGGCCCACGCGCTCCACGCCCAGCA 296
Sample_25-2      GCGAATGCCCGCCCAACTCGAAGAAGCCGTCCTGCCGGCCCACGCGCTCCACGCCCAGCA 292
Sample_33-2      GCGAATGCCCGCCCAGCTCGAAGAAGCCGTCCTGCCGGCCCACGCGCTCCACGCCCAGCA 295
Sample_15-2      GCGAATGCCCGCCCAGCTCGAAGAAGCCGTCCTGCCGGCCCACGCGCTCCACGCCCAGCA 276
Sample_30-2      GCGAATGCCCGCCCAGCTCGAAGAAGCCGTCCTGCCGGCCCACGCGCTCCACGCCCAGCA 307
Delftia          GCGAATGCCCGCCCAGCTCGAAGAAGCCGTCCTGCCGTCCCACGCGCTCCACGCCCAGCA 360
Sample_9-1       GCGAATGCCCGCCCAGCTCGAAGAAGCCGTCCTGCCGGCCCACGCGCTCCACGCCCAGCA 286
Sample_25-1      GCGAATGCCCGCCCAGCTCGAAGAAGCCGTCCTGCCGGCCCACGCGCTCCACGCCCAGCA 292
                 ********** **** ** ***************** ******** *************

Sample_12-2      CGTCCGCCCAGATCTGCGCCAGCGTTTCTTCCAACTCGCCCTGTGGTGCCTCGTATTGCT 377
Sample_17-3      CGTCCGCCCAGATCTGCGCCAGCGTTTCTTCCAACTCGCCCTGTGGTGCCTCGTATTGCT 378
Sample_15-1      CGTCCGCCCAGATCTGCGCCAGCGTTTCTTCCAACTCGCCCTGTGGTGCCTCGTATTGCT 356
Sample_26-1      CGTCCGCCCAGATCTGCGCCAGCGTTTCTTCCAACTCGCCCTGTGGTGCCTCGTATTGCT 355
Sample_1-3       CCTCGGCCCAGATCTGCGCCAGCGTTTCCTCCAGTTCTCCCTGCGGTGCCTCGTATTGCT 356
Sample_25-2      CCTCGGCCCAGATCTGCGCCAGCGTTTCTTCTAGTTCTGCCTGCGGTGCCTCGTATTGCT 352
Sample_33-2      CCTCGGCCCAGATCTGCGCCAGCGTTTCTTCCAGTTCTCCTTGCGGTGCCTCGTATTGCT 355
Sample_15-2      CCTCGGCCCAGATCTTCGCCAGCGTTTCTTCGAGTTCACCTTGCGGTGCCTCGTATTCCT 336
Sample_30-2      CCTCGGCCCAGATCTTCGCCAGCGTTTCTTCGAGTTCACCTTGCGGTGCCTCGTATTCCT 367
Delftia          CCTCGGCCCAGATCTGCGCCAGCGTTTCTTCGAGTTCACCTTGCGGTGCCTCGTATTCCT 420
Sample_9-1       CCTCGGCCCAGATCTGCGCCAGCGTTTCTTCCAGTTCACCTTGCGGTGCCTCGTATTCCT 346
Sample_25-1      CCTCGGCCCAGATCTGCGCCAGCGTTTCTTCCAGTTCACCTTGCGGTGCCTCGTATTCCT 352
                 * ** ********** ************ ** *  ** * ** ************* **

Sample_12-2      GGGCGCTGACCATCTCCGGCTCGGGCAGCGCCTTGCGGTCCACCTTGCCGTTGGCCGTCA 437
Sample_17-3      GGGCGCTGACCATCTCCGGCTCGGGCAGCGCCTTGCGGTCCACCTTGCCGTTGGCCGTCA 438
Sample_15-1      GGGCGCTGACCATCTCCGGCTCGGGCAGCGCCTTGCGGTCCACCTTGCCGTTGGCCGTCA 416
Sample_26-1      GGGCGCTGACCATCTCCGGCTCGGGCAGCGCCTTGCGGTCCACCTTGCCGTTGGCAGTCA 415
Sample_1-3       GGGCACTGACCATCTCCGGCTCGGGCAGCGCCTTGCGGTCCACCTTGCCGTTGGCCGTCA 416
Sample_25-2      GCGCACTCAGCATCTCCGGCTCGGGCAGCGCCTTGCGGTCCACCTTGCCGTTGGCCGTCA 412
Sample_33-2      GGGCACTCACCATCTCCGGCTCGGGCAGCGCCTTGCGGTCCACCTTGCCGTTGGCCGTCA 415
Sample_15-2      GCGCACTCGCCATCTCCGGCTCGGGCAGCGCCTTGCGGTCCACCTTGCCATTGGCTGTCA 396
Sample_30-2      GCGCACTCGCCATCTCAGGCTCGGGCAGCGCCTTGCGGTCCACCTTGCCGTTGGCTGTCA 427
Delftia          GCGCACTCGCCATCTCCGGCTCGGGCAGCGCCTTGCGGTCCACCTTGCCGTTGGCCGTCA 480
Sample_9-1       GCGCACTCACCATCTCCGGCTCGGGCAGCGCCTTGCGGTCCACCTTGCCGTTGGCCGTCA 406
Sample_25-1      GCGCACTCACCATCTCCGGCTCGGGCAGCGCCTTGCGGTCCACCTTGCCGTTGGCCGTCA 412
                 * ** ** ****** ******************************** ***** ****

Sample_12-2      GCGGCAGGGCGTCAAGCACGACGATGGCCGAGGGCACCATGTAGTCGGGCAGCGACTGGC 497
Sample_17-3      GCGGCAGGGCGTCAAGCACGACGATGGCCGAGGGCACCATGTAGTCGGGCAGCGACTGGC 498
Sample_15-1      GCGGCAGGGCATCGAGCACGACGATGGCCGAGGGCACCATGTAGTCGGGCAGCGCCTGGC 476
Sample_26-1      AAGGCAGGGCATCGAGCACGACGATGGCCGAGGGCACCATGTAGTCGGGCAGCACATGGC 475
Sample_1-3       AAGGCAGGGCATCGAGCACGACGATGGCCGAGGGCACCATGTAGTCGGGCAGCGCATGGC 476
Sample_25-2      AAGGCAGGGCATCGAGCACGACGATGGCCGAGGGCACCATGTAGTCGGGCAGCGCCTGGC 472
Sample_33-2      AAGGCAGGGCATCGAGCACGACGATGGCCGAGGGCACCATGTAGTCGGGCAGCGCCTGGC 475
Sample_15-2      GCGGCAGGGCTTCGAGCACGACGATGGCCGAGGGCACCATGTAGTCGGGCAGCGCCTGGC 456
Sample_30-2      GCGGCAGGGCTTCGAGCACGACGATGGCCGAGGGCACCATGTAGTCGGGCAGCGCCTGGG 487
Delftia          AAGGCAGGGCATCGAGCACGACGATGGCCGAGGGCACCATGTAGTCGGGCAGCGCCTGGC 540
Sample_9-1       AAGGCAGGGCATCGAGCACGACGATGGCCGAGGGCACCATGTAGTCGGGCAGTACCTGGC 466
Sample_25-1      AAGGCAGGGCATCGAGCACGACGATGGCCGAGGGCACCATGTAGTCGGGCAGTACCTGGC 472
                   ******** ** **************************************    ***

Sample_12-2      CCAGCCGCTGCTTGAGCTGGCTTTCCTCCACCGCGTCACGCAGGGAGACATAGGCGATCA 557
Sample_17-3      CCAGCCGCTGCTTGAGCTGGCTTTCCTCCACCGCGTCACGCAGGGAGACATAGGCGATCA 558
Sample_15-1      CCAGGCGCTGCTTGATCTGGCTTTCCTCCACCGCGTCACGCAGGGAGACATAGGCGATCA 536
Sample_26-1      CCAGGCGCTGCTTGATCTGACTTTCCTCCATCGCGTCATTCAGGGAGACATAGGCGATCA 535
Sample_1-3       CCAGGCGCTGCTTGATCTGGCTTTCCTCCACCGCGTCACGCAGGGAGACATAGGCGATCA 536
Sample_25-2      CCATGCGCTGCTTGATCTGGCTTTCCTCCACCGCGTCACGCAAGGAGACATAGGCGATCA 532
Sample_33-2      CCAGGCGCTGCTTGATCTGGCTTTCCTCCACCGCGTCACGCAGGGAGACATAGGCGATCA 535
Sample_15-2      CCAGCCGCTGCTTGAGCTGGCTTTCCTCCACCGCGTCACGCACGGAGACATAGGCGATCA 516
Sample_30-2      CCAGCCGCTGCTTGAGCTGGCTTTCCTCCACCGCGTCACGCAGGGAGACATAGGCGATCA 547
Delftia          CCAGGCGCTGCTTGAGCTGGCTTTCCTCCACCGCGTCACGCAGGGAGACATAGGCGATCA 600
Sample_9-1       CCAGGCGCTGCTTGAGCTGGCTTTCCTCCACCGCGTCACGCAGGGAGACATAGGCGATCA 526
Sample_25-1      CCAGGCGCTGCTTGAGCTGACTTTCCTCCACCGCGTCACGCAGGGAGACATAGGCGATCA 532
                 *** ********** *** ********** *******  ** *****************

Sample_12-2      GCCTTGCGCCCTCCTTGGCCAA-AACCACGGCCCCGCGCACCTCGGGCTGGGCCAGCAGC 616
Sample_17-3      GCCTTGCGCCCTCCTTGGCCAA-AACCACGGCCCCGCGCACCTCGGGCTGGGCCAGCAGC 617
Sample_15-1      GCCTTGCGCCCTCCTTGGCCAG-CACCACCGCCTCGCGCACCTCGGGCTGGGCCAGCAGC 595
Sample_26-1      GTCTTGCACCCTCCTTGG------------------------------------------ 553
Sample_1-3       GCCTTGCGCCCTCCTTGGCCAAACACCACCGCCTCGCGCACCTCGGGCTGGGCCAGCAGC 596
Sample_25-2      GCCTTGCGCCCTCCTTGGCCAA-AACCACGGCCTCGCGCACCTCGAGCTGGGCCAGCAGC 591
Sample_33-2      GCCTTGCACCCTCCTTGGCCAA-AACCACCGCCTCGCGCACCTCGGGCTGGGCCAGCAGC 594
Sample_15-2      GCCTTGCACCATCCTTGGCCAA-AACCACGGCCTCGCGCACCTCGGTCTGGGCCAGCAGC 575
Sample_30-2      GCCTTGCACCATCCTTGGCCAA-AACCACGGCCTCGCGCACCTCGGGCTGGGCCAGCAGC 606
Delftia          GCCTTGCACCCTCCTTGGCCAA-AACCACGGCCTCGCGCACCTCGGGCTGGGCCAGCAGT 659
Sample_9-1       GCCTTGCACCCTCCTTGGCCAG-CACCACGGCCTCGCGCACCTCGGGCTGGGCCAGC--- 582
Sample_25-1      GCCTTGCACCCTCCTTGGCCAG-CATCACGGCCTCGCGCACCTCGGGCTGGGCCAGC--- 588
                 * ***** ** *******                                         

Sample_12-2      TGCGACTGCACCTCGCCCAGCTCGATGCGGAAGCCCCGGATCTTGACCTGCTGGTCGGCA 676
Sample_17-3      TGCGACTGCACCTCGCCCAGCTCGATGCGGAAGCCCCGGATCTTGACCTGCTGGTCGGCA 677
Sample_15-1      TGCGACTGCACCTCGCGCAGCTCGATACGGAAGCCCCGGATCTTGACCTGCTGGTCG--- 652
Sample_26-1      ------------------------------------------------------------ 553
Sample_1-3       TGCGACTGCACCTCGCCCAGCTCGATGCGGAAGCCCCGGATCTTGACCTGCTGGTCGGCA 656
Sample_25-2      TGCGACTGCACCTCGCCCAGCTCGATGCGGAAGCCCCGGATCTTGACCTGCTGGTCGGCA 651
Sample_33-2      TGCGACTGCACCTCGCCCAGCTCGATGCGGAAGCCCCGGATCTTGACCTGCTGGTCGGCA 654
Sample_15-2      TGCGACTGCACCTCGCCCAGCTCGATGCGGAAGCCCCGGATCTTGACCTGCTGG------ 629
Sample_30-2      TGCGACTGCACCTCGCCCAGTTCGATGCGGAAGCCCCGGATCTTGACCTGCTGGTCGGCA 666
Delftia          TGCGACTGCACCTCGCCCAGCTCGATGCGGAAGCCCCGGATCTTGACCTGCTGGTCGGCA 719
Sample_9-1       ------------------------------------------------------------ 582
Sample_25-1      ------------------------------------------------------------ 588
                                                                            

Sample_12-2      CGGCCC-AGTATTCAAGTTCGCCCTGGGCACTCCAGCGCACCAGTC-GCCCGTGCGGTAC 734
Sample_17-3      CGGCCCACGTATTCAAGTTCGCCCTGGGCACTCCAGCGCACCAGTC-GCCCGTGCGGTAC 736
Sample_15-1      ------------------------------------------------------------ 652
Sample_26-1      ------------------------------------------------------------ 553
Sample_1-3       CGACCCAG-TATTCGAGTTCGCCCTGTGCACTCCAGCGCACCAGGTCGCCCGTGCGG-TA 714
Sample_25-2      C----------------------------------------------------------- 652
Sample_33-2      CGACCCAG-TATTCGAGTTCGCCCTGTGCACTCCAGCGCACCAGTCGCCCGTGCGGT-AC 712
Sample_15-2      ------------------------------------------------------------ 629
Sample_30-2      CGGCCCACGTATTCGAGTTCGCCCTGAGTGTTCCAGCGCACCAGATCGCCCGTGCGGTAC 726
Delftia          CGACCCAGGTATTCGAGTTCGCCCTGTGCACTCCAGCGCACCAGGTCGCCCGTGCGGTAC 779
Sample_9-1       ------------------------------------------------------------ 582
Sample_25-1      ------------------------------------------------------------ 588
                                                                            

Sample_12-2      AGGCGCTCGCCCGTCTCGCTGACGGG---------------------------------- 760
Sample_17-3      AGGCGCTCGCCCGTCTCGCTGAACGGGTTGG----------------------------- 767
Sample_15-1      ------------------------------------------------------------ 652
Sample_26-1      ------------------------------------------------------------ 553
Sample_1-3       CAGCGCTCGCCCGTCTCGCTGAACGGGTTG------------------------------ 744
Sample_25-2      ------------------------------------------------------------ 652
Sample_33-2      AGGCGCTCGCCCGTCTCGCTGACGGGTTGGC----------------------------- 743
Sample_15-2      ------------------------------------------------------------ 629
Sample_30-2      AGGCGCTCGCCCGTCTCGCTGAACGGG--------------------------------- 753
Delftia          AGGCGCTCGCCCGTCTCGCTGAACGGGTTGGCAATGAAGCGCTCTGCCGTCAGTCCTTGC 839
Sample_9-1       ------------------------------------------------------------ 582
Sample_25-1      ------------------------------------------------------------ 588
                                                                            

Sample_12-2      ------------------------------------------------------------ 760
Sample_17-3      ------------------------------------------------------------ 767
Sample_15-1      ------------------------------------------------------------ 652
Sample_26-1      ------------------------------------------------------------ 553
Sample_1-3       ------------------------------------------------------------ 744
Sample_25-2      ------------------------------------------------------------ 652
Sample_33-2      ------------------------------------------------------------ 743
Sample_15-2      ------------------------------------------------------------ 629
Sample_30-2      ------------------------------------------------------------ 753
Delftia          CTGTTGAGATAGCCGCGTGCCAAGCCCTCGCCCGCCACATACAACTCTCCCGCCACACCC 899
Sample_9-1       ------------------------------------------------------------ 582
Sample_25-1      ------------------------------------------------------------ 588
                                                                            

Sample_12-2      ------------------------------------------------------------ 760
Sample_17-3      ------------------------------------------------------------ 767
Sample_15-1      ------------------------------------------------------------ 652
Sample_26-1      ------------------------------------------------------------ 553
Sample_1-3       ------------------------------------------------------------ 744
Sample_25-2      ------------------------------------------------------------ 652
Sample_33-2      ------------------------------------------------------------ 743
Sample_15-2      ------------------------------------------------------------ 629
Sample_30-2      ------------------------------------------------------------ 753
Delftia          TGCGGCAGCAGGTTCAGGCTGCCGTCGAGCACGTACAGGCCCAGGTCCGGAATCGCCACG 959
Sample_9-1       ------------------------------------------------------------ 582
Sample_25-1      ------------------------------------------------------------ 588
                                                                            

Sample_12-2      ------------------------------------------------------------ 760
Sample_17-3      ------------------------------------------------------------ 767
Sample_15-1      ------------------------------------------------------------ 652
Sample_26-1      ------------------------------------------------------------ 553
Sample_1-3       ------------------------------------------------------------ 744
Sample_25-2      ------------------------------------------------------------ 652
Sample_33-2      ------------------------------------------------------------ 743
Sample_15-2      ------------------------------------------------------------ 629
Sample_30-2      ------------------------------------------------------------ 753
Delftia          CCCACGGGGCTGCGTCCGCCGTCCAGATCCTTCTGGATGATCTCCCGGTACGTCACATGC 1019
Sample_9-1       ------------------------------------------------------------ 582
Sample_25-1      ------------------------------------------------------------ 588
                                                                            

Sample_12-2      ----------------------------------- 760
Sample_17-3      ----------------------------------- 767
Sample_15-1      ----------------------------------- 652
Sample_26-1      ----------------------------------- 553
Sample_1-3       ----------------------------------- 744
Sample_25-2      ----------------------------------- 652
Sample_33-2      ----------------------------------- 743
Sample_15-2      ----------------------------------- 629
Sample_30-2      ----------------------------------- 753
Delftia          ACCGTGGTCTCGGTGATGCCGTACATGTTGATGAG 1054
Sample_9-1       ----------------------------------- 582
Sample_25-1      ----------------------------------- 588
