# Supplementary material for: Catalyzing rapid discovery of gold-precipitating bacterial lineages with university students
Source: PeerJ. 2020 Apr 14;8:e8925. doi: 10.7717/peerj.8925 (PMC7164421; doi:10.7717/peerj.8925)
Supplement: Supplemental Information 8 [file peerj-08-8925-s008.docx]

**Supplemental Data S7:** Alignment of *D. acidovorans* strain ANG1 and environmental samples

Delftia          AGATGTCCTGGATGTTGGCTGCGCCACCGGGCACCGCCTGCGCAATGCGAGCAATCTCCT 60
Sample_7-1       ------------------------------------------------------------ 0
Sample_18-1      ------------------------------------------------------------ 0
Sample_23-2      ---------------------------------------------ATGCGCGCATCTCCT 15
Sample_24-1      ------------------------------------------------------------ 0
                                                                            

Delftia          CCTCATCCAGCGCCACCAGGGTCAGCATGTCCGGCGTGATCGCCGTGCAGCCTTCGGGGA 120
Sample_7-1       -----TCCAGCGCCACCAGGGTCAGCATGTCCGGCGTGATCGCCATGCAGCCTTCGGGGA 55
Sample_18-1      -------------------------TAGTCCGTGCGTGATCGCCGTGCAGCCTTCGGGGA 35
Sample_23-2      CCTCATCCAGCGCCACCAGGGTCAGCATGTCCGGCGTGATCGCCGTGCAGCCTTCGGGGA 75
Sample_24-1      ---------------CCAGGGTCAGCATGTCCGGCGTGATCGCCGTGCAGCCTTCGGGGA 45
                                           * * *********** ***************

Delftia          TGCCGTTGGGCGGCACATCGATCTCGCCGGCCACCTGCTCCCCTTGGTCCCCCTGCTGCG 180
Sample_7-1       TGCCGTTGGGCGGCACATCGATCTCGCCGGCCACCTGCGCCCCTTGCTCACCCTGCTGAT 115
Sample_18-1      TGCCGTTGGGCGGCACATCGATCTCGCCGGCCACCTGCTCCCCTTGCTCACCCTGCTGCT 95
Sample_23-2      TGCCGTTGGGTGGCACATCGATCTCGCCGGCCACCTGCTCACCCTGCTGCTCCTGCTGTT 135
Sample_24-1      TGCCGTTGGGTGGCACATCGATCTCGCCGGCCACCTGCTCACCCTGCTGCTCCTGCTGTT 105
                 ********** *************************** * ** ** *   *******

Delftia          CCTGCAACACTGCCTGCGCAAACTCCGCCAGCCTCGGGTGCTGGAACAGCGTGCGCACCT 240
Sample_7-1       CCTGCCGCACCGCCTGCACAAACTCTGCCAACCTCGGGTGCTGGAACAGCTTGAGCACCT 175
Sample_18-1      CCTGCTGCACCGCCTGCGCAAACTCCGCCAGCCTCGGGTGCTGGAACAGCGTGCGCACCT 155
Sample_23-2      CCTCCAATACCGCCTGCGCAAACTCCGCCAGCCTCGGATGCTGGAACAGCGTGCGCACCT 195
Sample_24-1      CCTCCAATACCGCCTGCGCAAACTCCGCCAGCCTCGGATGCTGGAACAGCGTGCGCACCT 165
                 *** * ** ****** ******* **** ****** ************ ** ******

Delftia          GCACGCGCAGGCCCTGGGCGCGCACGCGCTCCAGCAGGCCCAGGGCCAGCAGCGAATGCC 300
Sample_7-1       GGACGCGCAGGCCCCGGGCGCGCAGGCGCTCCACCAGGCCCAGGGCGAGCAGGGAATGCC 235
Sample_18-1      GCACGCGCAGGCCCTGGGCGCGCACGCGCTCCAGCAGGCCCAGGGCGAGCAGCGAATGCC 215
Sample_23-2      GCACGCGCAGGCCCTGGGCGCGCACGCGCTCCAGCAGGCCCAGGGCCAGCAGCGAATGCC 255
Sample_24-1      GCACGCGCAGGCCCTGGGCGCGCACGCGCTCCAGCAGGCCCAGGGCCAGCAGCGAATGCC 225
                 * ************ ********* ******** ************ ***** *******

Delftia          CGCCCAGCTCGAAGAAGCCGTCCTGCCGGCCCACGCGATCCACGCCCAGCACCTCGGCCC 360
Sample_7-1       CGCCCAGCTCGAACAAGCCGTCCTGCCGGCCCACGCGCTCCACGCCCAGCACCTCGGCCC 295
Sample_18-1      CGCCCAGCTCGAAGAAGCCGTCCTGCCGGCCCACGCGCTCCACGCCCAGCACCTCGGCCC 275
Sample_23-2      CGCCCAGATCGAAGAAGCCGTCCTGCCGGCCCACGCGCTCCACGCCCAGCACCTCGGCCC 315
Sample_24-1      CGCCCAGATCGAAGAAGCCGTCCTGCCGGCCCACGCGCTCCACGCCCAGCACCTCGGCCC 285
                 ******* ***** *********************** **********************

Delftia          AGATCGTCGCCAGCGTTTCCTCCAACTCGCCCTGCGGTGCCTCGTATTGCTGGGCGCTGA 420
Sample_7-1       ACATCTGCGCCATCGTTTCCTCCAGCTCTCCCTGCGGTGCCTCGTATTGCTGGGAGCTGA 355
Sample_18-1      AGATCTGCGCCAGCGTTTCCTCCAGTTCTCCCTGCGGTGCCTCGTATTGCTGGGCGCTCA 335
Sample_23-2      AGATCTGCGCCAGCGTTTCCTCCAACTCGCCCTGCGGTGCCTCATATTGCTGGGCGCTGA 375
Sample_24-1      AGATCTGCGCCAGCGTTTCCTCCAACTCGCCCTGCGGTGCCTCATATTGCTGGGCGCTGA 345
                 * *** ***** ***********  ** ************** ********** *** *

Delftia          TCATCTCCGGCTCGGGCAGCGCCTTGCGGTCCACCTTGCCGTTGGCTGTCAGCGGCAGGG 480
Sample_7-1       GCATCTCCGGCTCGGGCAGCGCCTTGCGGGCCATCTTGCCGTTGGTGGTCAAAGGCAGGG 415
Sample_18-1      CCATCTCCGGCTCGGGCAGCGCCTTGCGGTCCACCTTGCCGTTGGCCGTCAAAGGCAGGG 395
Sample_23-2      CCATCTCCGGCTCGGGCAGCGCCTTGCGGTCCACCTTGCCGTTGGCGGTCAAAGGCAGGG 435
Sample_24-1      CCATCTCCGGCTCGGGCAGCGCCTTGCGGTCCACCTTGCCGTTGGTGGTCAAAGGCAGGG 405
                  **************************** *** ***********  **** *******

Delftia          CTTCGAGCACGACGATGGCCGAGGGCACCATGTAGTCGGGCAGCGCCTGGCCCAGGCGCT 540
Sample_7-1       CGTCGAGGACGACGATGGCCAAGGGCACCATGTAGTCCGGGAGCACCTGGACCAGTCGTC 475
Sample_18-1      CATCGAGCACGACGATGGCCGAGGGCACCATGTAGTCGGGCAGTACCTGGCCCAGCCGGT 455
Sample_23-2      CATCGAGCACGACGATGGCCGAGGGCACCATGTAGTCGGGCAGTATCTCGCCCAGCCGGT 495
Sample_24-1      CATCGAGCACGACGATGGCCGAGAGCACCATGTAGTCGGGCAGTATCTCGCCCAGCCGGT 465
                 * ***** ************ ** ************* ** **   ** * **** **

Delftia          GCTTGAGCTGGCTTTCCTCCACCGCGTCACGCAGGGAGACATAGGCGATCAGCCTTGC-- 598
Sample_7-1       CCTTGAGCTGACCCTCTGCGATTTCTGCATTCAGCGACACATAGGCGATCAATCTGATAA 535
Sample_18-1      CCTTGAGCTGACTGTCTTCGATTTCTGCATTCAGCGACACATAGGCGATAAGTCTGACAC 515
Sample_23-2      CCTTGAGCAGACCCTCTGCGATTTCTGCATTCAGCGACACATAGGCGATCAGTCTGGCAC 555
Sample_24-1      CCTTGAGCAGACCCTCTGCGATTTCTGCATTCAGCGACACATAGGCGATCAGTCTGGCAC 525
                  ******* * * ** * *   * ** *** ** *********** *  **

Delftia          -------ACCCTCCTTGGCCAGCACCACCGCCTCACGCAC-TTCAGGCTGGGCCAGCAGC 650
Sample_7-1       CGCCCGCGCCCTCCTTGGCCAGCACCACTGCCTCGCGCACCTTCGGGCTGGGCCAGCAGT 595
Sample_18-1      CGCCTGCGCCCTCCTTGGCCAGCACCACCGCCTCACGCACC-TCGGGCTGGGCCAGCAGC 574
Sample_23-2      CGCCCGCCCCCTCCTTGGCCAGCACCACCGCCTCACGCACT-TCAAGCTGGGCCAGCAGC 614
Sample_24-1      CGCCCGCCCCCTCCTTGGCCAGCACCACCGCCTCACGCACT-TCAGGCTGGGCCAGCAGT 584
                         ******************** ***** *****  ** *************

Delftia          TGCGACTGCACCTCGCCCAGTTCGATGCGGAAGCCCCGGATCTTGACCTGCTGGTCGGCA 710
Sample_7-1       TGCGACTGCACCTCGCCCAGCTCGATGCGGAAGCCCCGGATCTTGACCTGCTGGTCGGCA 655
Sample_18-1      TGCGACTGCACCTCGCCCAGCTCGATGCGGAAGCCCCGGATCTTGACCTGCTGGTCGG-- 632
Sample_23-2      TGCGACTGCACCTCGCCCAGCTCGATGCGGAAGCCCCGGATCTTGACCTGCTGGTCGGCA 674
Sample_24-1      TGCGACTGCACCTCGCCCAGCTCGATGCGGAAGCCCCGGATCTTGACCTGCTGGTCGGCA 644
                 ******************** ************************************* 

Delftia          CGACCCAGGTATTCGAGTTCGCCCTGTGCACTCCAGCGCACCAGATCGCCCGTGCGGTAC 770
Sample_7-1       CGGCCCAG-TATTCGAGTTCGCCCTGAGCGTTCCAGCGCACCAAGTCGCCCGTGCGGTAC 714
Sample_18-1      ------------------------------------------------------------ 632
Sample_23-2      CGGTCCAG-TATTCCAGTTCGCCCTGAGCGTTCCAGCGCACCAGATCGCCCGTGCGGTAC 733
Sample_24-1      CGGCCCAGGTATTCGAGTTCGCCCTGAGCGTTCCAGCGCACCAGATC------------- 691
                                                                            

Delftia          AGGCGATCGCCTGCCTGTGTGAAGGGATTGGCGATAAAGCGCTCGGCGCTCAGGCCAGCG 830
Sample_7-1       A----------------------------------------------------------- 715
Sample_18-1      ------------------------------------------------------------ 632
Sample_23-2      AGACGCTCGCC------------------------------------------------- 744
Sample_24-1      ------------------------------------------------------------ 691
                                                                            

Delftia          CGATTCAGGTAGCCGCGTGCCAGTCCTTCGCCCGCCACGTACAGCTCCCCGGCCACGCCC 890
Sample_7-1       ------------------------------------------------------------ 715
Sample_18-1      ------------------------------------------------------------ 632
Sample_23-2      ------------------------------------------------------------ 744
Sample_24-1      ------------------------------------------------------------ 691
                                                                            

Delftia          TGCGGCAGCAGGTTCAGGCTGCCATCGAGCACGTACAGGCCCAGGTCCGGAATAGCCACG 950
Sample_7-1       ------------------------------------------------------------ 715
Sample_18-1      ------------------------------------------------------------ 632
Sample_23-2      ------------------------------------------------------------ 744
Sample_24-1      ------------------------------------------------------------ 691
                                                                            

Delftia          CCAACGGGGCTGCGGCCACCATCCAAATCCGCCTTGGTGATCTGACGGTACGTCACATGC 1010
Sample_7-1       ------------------------------------------------------------ 715
Sample_18-1      ------------------------------------------------------------ 632
Sample_23-2      ------------------------------------------------------------ 744
Sample_24-1      ------------------------------------------------------------ 691
                                                                            

Delftia          ACCGTGGTCTCGGTGATGCCGTACATGTTGATGAG 1045
Sample_7-1       ----------------------------------- 715
Sample_18-1      ----------------------------------- 632
Sample_23-2      ----------------------------------- 744
Sample_24-1      ----------------------------------- 691
