# Supplementary material for: Catalyzing rapid discovery of gold-precipitating bacterial lineages with university students
Source: PeerJ. 2020 Apr 14;8:e8925. doi: 10.7717/peerj.8925 (PMC7164421; doi:10.7717/peerj.8925)
Supplement: Supplemental Information 10 [file peerj-08-8925-s010.docx]

**Supplemental Data S9:** Alignment of *D. acidovorans* strain RAY209 and environmental samples

Sample_7-2       ------------------------------------------------------------ 0
Delftia          AGATGTCCTGGATGTTGGCTGCGCCACCGGGCACCGCCTGCGCAATGCGTGCAATCTCAT 60
Sample_30-3      ------------------------------------------------------------ 0
                                                                            

Sample_7-2       -----TCCAGCGCCACCAGGGTCAGCATGTCCGGCGTGATCACCGTGCAGCCTTCGGGGA 55
Delftia          CCTCATCCAGCGCCACCAGGGTCAGCATGTCCGGCGTGATCTCCGTGCAGCCTTCGGGGA 120
Sample_30-3      --------AGCGCCACCAGGGTCAGCATGTCCGGCGTGATCACCGTGCAGCCTTCGGGGA 52
                         ********************************* ******************

Sample_7-2       TGCCGTTGGGTGGCACATCGATCTCGCCGGCCACCTGCTCACCTTGCTGATCCTGCTGTT 115
Delftia          TGCCGTTGGGCGGCACATCGATCTCGCCGGCCACCTGCGCCCCTGGCTCACCCTGCTGAT 180
Sample_30-3      TGCCGTTGGGTGGCACATCGATCTCGCCGGCCACCTGCTCACCCTGCTGCTCCTGCTGTT 112
                 ********** *************************** * **  *** ******* *

Sample_7-2       CCTGCTGTTCCTCCTGTACAAACTGCGCCAACTTCGGATGCTGGAAAAGCTGGAACACCT 175
Delftia          CCTGCAGCACCGCCTGCGCAAACTCCGCCAACCTCGGGTGCTGGAACAGCGTGCGCACCT 240
Sample_30-3      CCTCCAGTACCGCCTGCGCAAACTCCGCCAGCCTCGGATGCTGGAACAGCGTGCGCACCT 172
                 *** * * ** **** ****** ***** * **** ******** ***  * *****

Sample_7-2       GGCCCCCCTGGACCTGGGCGCGCAGGGGCTCCACCAGGTCCAGGGGGAGCAGGGAAAGCC 235
Delftia          GCACGCGCAGGCCCTGGGCGCGCACGCGCTCCAGCAGGCCCAGGGCGAGCAGAGAATGCC 300
Sample_30-3      GCACGCGCAGGCCCTGGGCGCGCACGCGCTCCAGCAGGCCCAGGGCGAGCAGCGAATGCC 232
                 * * * * ** ************ * ****** **** ****** ****** *** ***

Sample_7-2       CCCAATGCTCGAACAAGTCGTCCAGCCCTCCCTGGCGCTCCACGCCCACAACGTCCGCCA 295
Delftia          CGCCCAGCTCGAAGAAGCCGTCCTGCCGTCCCACGCGCTCCACGCCCAGCACCTCGGCCC 360
Sample_30-3      CGCCCAGCTCGAAGAAGCCGTCCTGCCGTCCCACGCGCTCCACGCCCAGAACGTCCGCCC 292
                 * * ******* *** ***** *** ****  ************** ** ** ***

Sample_7-2       AGATCTTCCACATCTTTTCTTCCAGTTCTTCTTGCTGTGCCTGGTATTCCTGCTATTGCT 355
Delftia          AGATCTTCGCCAGCGTTTCTTCGAGTTCACCTTGCGGTGCCTCGTATTCCTGCGCACTCG 420
Sample_30-3      AGATCTTCGCCAGCGTTTCTTCGAGTTCACCTTGCGGTGCCTCGTATTCCTGCGCACTCG 352
                 ******** ** * ******* *****  ***** ****** ********** *

Sample_7-2       GCATCTTCGGCTTGTGCAGCTCGTTGAGCTCCTTGTTGCCCATGGTGGTCTTAGGCATGG 415
Delftia          CCATCTCCGGCTCGGGCAGCGCCTTGCGGTCCACCTTGCCGTTGGCAGTCAGCGGCAGGG 480
Sample_30-3      CCATCTCCGGCTCGGGCAGCGCCTTGCGGTCCACCTTGCCGTTGGCTGTCAGCGGCAGGG 412
                  ***** ***** * ***** * *** * ***   ***** *** *** **** **

Sample_7-2       CTTCCAGCACAACGATGGCCAAAAGCACCGAGTAGTCCGTGAACGCCTGGACCACACGTC 475
Delftia          CGTCAAGCGCGACGATGGCCGAGGGCACCATGTAGTCGGGCAGCGCCTGGCCCAGCCGGT 540
Sample_30-3      CGTCGAGCACGACGATGGCCGAAGGCATCATGTAGTCGGGCAGCGCATGGCCCAGCCGGT 472
                 * ** *** * ********* *  *** * ****** * * *** *** ***  **

Sample_7-2       CCTTGAGCTGATTGATCTGGATTTCCGCATTCACGTACACATAGGAGATATATCTGATCA 535
Delftia          CCTTGAGCCAACCGTCTTCGACCGGCGAATTCAGCGAGACATAGGCCACCAATCGCGCCC 600
Sample_30-3      CCTTGAGCAAACCGTCTTCGGCTGGCGAATTCAGCGAGACATAGGCCACCAATCGCGCCC 532
                 ******** * * * *     ** ***** * ******* * ***    *

Sample_7-2       CGCCTGCCCCCTCCTTGGCCAGCACCACCGCCTCGCGCACCTCGGGCTGGGCCAGCAGCT 595
Delftia          CGCCTGTGCTTTCCTTGGCCAGCACCACGGCCTCGCGCACCTCGGGCTGGGCCAGCAGCT 660
Sample_30-3      CGCCTGCGCTTTGCTTGGCCAGCACCACCGCCTCACGCACTTCAGGCTGGGCCAGCAGTT 592
                 ****** * * *************** ***** ***** ** ************** *

Sample_7-2       GCGACTGCACCTCGCCCAGCTCGATGCGGAAGCCCCGGATCTTGACCTGCTGGTCGGCAC 655
Delftia          GCGACTGCACCTCGCCCAGCTCGATGCGGAAGCCCCGGATCTTGACCTGCTGGTCGGCAC 720
Sample_30-3      GCGACTGCACCTCGCCCAGCTCGATGCGGAAGCCCCGGATCTTCACCTGCTGGTCGGCAC 652
                 ******************************************* ****************

Sample_7-2       GGCCCAGTATTCG-AGTTCGCCCTGAGCA------------------------------- 683
Delftia          GGCCCAGGTATTCGAGTTCGCCCTGAGCGTTCCAGCGCACCAGATCGCCCGTGCGGTACA 780
Sample_30-3      GGCCCACGTATTCGAGTTCGCCCTGAGCGTTCCAGCGCACCAGATCGCCTGTGCGGTACA 712
                 ****** * **************                               

Sample_7-2       ------------------------------------------------------------ 683
Delftia          GGCGATCGCCTGCCTGTGTAAAGGGATTGGCGATGAAGCGCTCGGCGCTCAGACCGGCGC 840
Sample_30-3      GGCGCTCGCCG------------------------------------------------- 723
                                                                            

Sample_7-2       ------------------------------------------------------------ 683
Delftia          GGTTCAGGTAACCACGTGCCAGGCCCTCACCCGCCACATACAACTCTCCCGCCACGCCCT 900
Sample_30-3      ------------------------------------------------------------ 723
                                                                            

Sample_7-2       ------------------------------------------------------------ 683
Delftia          GCGGCAGCAGGTTCAGGCTGCCGTCGAGCACGTACAGGCCCAGGTCCGGAATCGCCACGC 960
Sample_30-3      ------------------------------------------------------------ 723
                                                                            

Sample_7-2       ------------------------------------------------------------ 683
Delftia          CCACGGGGCTGCGTCCGCCGTCCAGATCCTTCCGGGTGATCTCCCGGTACGTCACATGCA 1020
Sample_30-3      ------------------------------------------------------------ 723
                                                                            

Sample_7-2       ---------------------------------- 683
Delftia          CCGTGGTCTCGGTGATGCCGTACATGTTGATGAG 1054
Sample_30-3      ---------------------------------- 723
